# 1α,25(OH)_2_D_3_ ameliorates insulin resistance by alleviating γδ T cell inflammation via enhancing fructose-1,6-bisphosphatase 1 expression

**DOI:** 10.7150/thno.84645

**Published:** 2023-09-25

**Authors:** Peng Li, Ke Li, Wenhui Yuan, Yuqi Xu, Ping Li, Ruan Wu, Jingru Han, Zhinan Yin, Ligong Lu, Yunfei Gao

**Affiliations:** 1 Guangdong Provincial Key Laboratory of Tumor Interventional Diagnosis and Treatment, Zhuhai Institute of Translational Medicine, Zhuhai People's Hospital Affiliated with Jinan University, Jinan University, Zhuhai, 519000, Guangdong, China.; 2 The Biomedical Translational Research Institute, Faculty of Medical Science, Jinan University, Guangzhou, 510632, Guangdong, China.; 3 Department of Geriatrics, The Seventh Affiliated Hospital, Sun Yat-Sen University, Shenzhen, 518107, Guangdong, China.; 4 Department of Endocrinology, Guangdong Second Provincial General Hospital, Guangzhou, 510310, Guangdong, China.; 5 Anhui Provincial Center for Disease Control and Prevention, Hefei, 230601, Anhui, China.; 6 Department of Oncology, First Affiliated Hospital, Jinan University, Guangzhou, 510632, Guangdong, China.

**Keywords:** vitamin 1α, 25(OH)_2_D_3_, γδ T cell, fructose-1, 6-bisphosphatase 1, glycolysis, inflammation, insulin resistance

## Abstract

**Background:** Chronic inflammation caused by immune cells is the central link between obesity and insulin resistance. Targeting the inflammatory process is a highly promising method for reversing systemic insulin resistance.

**Methods:** Blood samples were prospectively collected from 68 patients with type 2 diabetes. C57BL/6J mice were fed either a high-fat diet (HFD) or normal chow (NC). We performed phenotypical and functional analyses of immune cells using flow cytometry. Vitamin D receptor (VDR) knockout γδ T cells were constructed using Cas9-gRNA targeted approaches to identify 1α,25(OH)_2_D_3_/VDR signaling pathway-mediated transcriptional regulation of fructose-1,6-bisphosphatase (FBP1) in γδ T cells.

**Results:** Serum vitamin D deficiency aggravates inflammation in circulating γδ T cells in type 2 diabetes patients. We defined a critical role for 1α,25(OH)_2_D_3_ in regulating glycolysis metabolism, protecting against inflammation, and alleviating insulin resistance. Mechanistically, 1α,25(OH)_2_D_3_-VDR promoted FBP1 expression to suppress glycolysis in γδ T cells, thereby inhibiting Akt/p38 MAPK phosphorylation and reducing inflammatory cytokine production. Notably, therapeutic administration of 1α,25(OH)_2_D_3_ restrained inflammation in γδ T cells and ameliorated systemic insulin resistance in obese mice.

**Conclusions:** Collectively, these findings show that 1α,25(OH)_2_D_3_ plays an important role in maintaining γδ T cell homeostasis by orchestrating metabolic programs, and is a highly promising target for preventing obesity, inflammation, and insulin resistance.

## Background

Epidemiological and observational studies have shown that serum vitamin D deficiency is associated with insulin resistance and increased risk of type 2 diabetes (T2D) 1-4. Previous studies have shown that vitamin D (its bioactive form 1α,25(OH)_2_D_3_) and the vitamin D receptor (VDR) are involved in the development of T2D, suggesting a potential therapeutic role for vitamin D in diabetes 5-8. Evidence from several intervention studies and meta-analyses has shown that vitamin D supplementation might have beneficial effects on glycemic control 3, 9-11, whereas some clinical trials and other meta-analyses have not supported the efficacy of vitamin D supplementation for diabetes prevention or therapy 12, 13. A possible explanation for these inconsistent conclusions is that the function of vitamin D in the prevention and treatment of diabetes is affected by individual obesity, metabolic disorders, and other factors.

Obesity increases the risk of multiple diseases including hypertension, cancer, insulin resistance, and T2D 14-16. Systemic chronic low-grade inflammation is a critical link between obesity and insulin resistance 17-20. The source of inflammatory cytokines was initially considered to be adipocytes, whereas immune cells in adipose tissue (AT), such as αβ T, NK, macrophage cells, and innate-like γδ T cells, in the case of obesity, could also trigger inflammation and aggravate insulin resistance 21-25. Blocking pro-inflammatory activity reduces the expression of cytokines (TNF, IL-6, IL-1β, IFN-γ, and IL-17) and ameliorates insulin resistance 17, 26, 27. After a long-term high-fat diet, tissue residual γδ T cells are the major source of IL-17a, which regulates adipogenesis and glucose metabolism in AT 28. These findings suggest that targeting the inflammatory signaling pathway of γδ T cells might be a potential way to reverse insulin resistance.

Obese donors exhibit a reduction in γδ T cells the peripheral blood, and the severity of obesity negatively correlates with the number of γδ T cells 29. Additionally, vitamin D deficiency was associated with an increased Body Mass Index (BMI) in studies of both diabetic and non-diabetic subjects 30. Insulin resistance mediated by vitamin D deficiency has been proposed to be related to obesity and chronic inflammation 31-33. However, the mechanisms by which vitamin D regulates cytokine production by γδ T cells in obesity and T2D have not been well defined. Both oxidative phosphorylation (OXPHOS) and glycolysis provide energy for proliferation, differentiation, and activation of immune cells 34-36. The metabolism enzymes in immune cells are critical for normal immunity responses 35, 37, 38. FBP1, a rate-limiting enzyme related to gluconeogenesis, mainly induces gluconeogenesis and suppresses glycolysis metabolism 39, suggesting that FBP1 might be involved in mediating inflammatory responses.

In this study, we detected the level of 25(OH)D_3_ in serum and found that serum 25(OH)D_3_ decreased as the BMI increased in T2D patients, and it was also inversely correlated with the expression of TNF-α and IFN-γ in circulating γδ T cells. Administration of 1α,25(OH)_2_D_3_ mediated weight loss, simultaneously reduced the expression of inflammatory cytokines in tissue residual γδ T cells, and ameliorated insulin resistance in HFD-induced obesity mouse models. Furthermore, 1α,25(OH)_2_D_3_-VDR promoted FBP1 expression to repress glycolysis, decrease the phosphorylation of Akt-p38 MAPK signaling, and reduce the production of TNF-α and IFN-γ. These findings suggest that 1α,25(OH)_2_D_3_ has the potential to treat obesity and insulin resistance.

## Methods

### Material details

All material name catalog numbers, manufacturers, cities, and nations are provided in the Supplementary Material Table S4 (Key Resources Table).

### Mice

C57BL/6J mice were purchased from the Charles River. Animal protocols were approved by the Institutional Animal Care and Use Committee of Jinan University. Male mice were housed in a temperature- and humidity-controlled, specific-pathogen-free animal facility at 25°C under a light-dark cycle with free access to water and food. For the diet study, mice were fed a 60% HFD (Research Diets) beginning at six weeks of age. The body weights of the mice were measured weekly.

### Human samples

This study was approved by the Institutional Review Board of Guangdong Second Provincial General Hospital. We recruited 68 individuals who had been diagnosed with type 2 diabetes. Information on the patients with T2D is provided in Table S1. All healthy donors (BMI, 18.5-23.9) were enrolled from the medical examination department of Guangdong Second Provincial General Hospital.

### PBMCs isolation

Peripheral blood mononuclear cells (PBMCs) of T2D patients and healthy donors (HD) were isolated from whole blood following the standard Ficoll-Paque-based (GE Healthcare) density gradient centrifugation protocol 40. Human sera were obtained by centrifugation before isolation of PBMCs.

### γδ T cell culture* in vitro*

Human PBMCs isolated from healthy donors were stimulated with 50 μM zoledronic acid monohydrate (ZOL) and 100 IU/mL recombinant human IL-2 in 24-well round-bottom microculture plates 41. γδ (Vδ2) T-cells were cultured in RPMI 1640 medium supplemented with 10% FBS. IL-2 was added every two days over a culture period of 10-15 days until Vδ2 T cells represented >90% of the total cell population. In some experiments, γδ T cells originating from PBMCs were purified by negative selection using an EasySep™ Human Gamma/Delta T Cell Isolation Kit. Unless mentioned otherwise, Vδ2 T cells used in all experiments were pretreated with 100 nM 1α,25(OH)_2_D_3_ (Enzo) or vehicle three times at 1-day intervals.

### Quantitative RT-PCR

Total RNA was isolated using the RNA simple Total RNA Kit, and cDNA was synthesized using the PrimeScript RT Reagent Kit according to the manufacturer's instructions. qPCR was carried out using SYBR Green PCR Master Mix according to the manufacturer's instructions. Relative mRNA expression was calculated using the 2^-ΔΔCt^ method; after normalization to β‐actin. The heatmap (Fig. 3f) was described as a fold change according to the following formula:



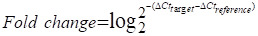



RNA levels were normalized to β‐actin mRNA levels. A list of the primer sequences is provided in Table S2.

### Mouse models treatment

WT mice were fed a high-fat diet (HFD) for 10-12 weeks to induce obesity. Mice were randomly divided into two groups for intraperitoneal (i.p.) injection of Calcitriol (0.01 mg/kg), 5 mg/kg MB05032 (MCE), 200 μg αTCRγ/δ (BioLegend), PBS alone, or a combination of 3-4 times at 5-day intervals.

### Glucose tolerance tests

Mice were fasted for 14-16 h before glucose tolerance tests. Glucose was administered by i.p. injection on the basis of body weight (1 g/kg), and blood glucose levels were detected at indicated times of 0, 30, 60, 90, and 120 minutes (min) using blood glucose strips. The blood glucose level at 0 min was defined as the fasting glucose level.

### Insulin tolerance tests

For the ITT test, mice were fasted for 2-4 h and i.p. injected with 0.75 U/kg insulin, and blood glucose levels were measured at 0, 30, 60, 90, and 120 min using blood glucose strips.

### Isolation of immune cells in adipose tissue

The epididymal white adipose tissue (eWAT) was dissected and weighed. The tissue was digested for 60 min at 37 ℃ in RPMI 1640 medium containing 1 mg/mL collagenase Ⅱ and 2% FBS. The tissue suspension was filtered through a 100 μm cell strainer and centrifuged at 600 g for 7 min to pellet the stromal vascular fraction (SVF) 42. Mouse SVF pellets were used for surface or intracellular cytokine staining.

### Histology

Mouse liver and adipose tissues (SCW and eWAT, respectively) were fixed in 4% paraformaldehyde overnight before sectioning. The tissues were stained with hematoxylin and eosin (H&E) and photographed under a microscope. A representative image from each group is shown in our study.

### ChIP-qPCR

1α,25(OH)_2_D_3_- or vehicle- pretreated Vδ2 T cells were re-stimulated with 1α,25(OH)_2_D_3_ for 12 h. Activated Vδ2 T cells were subjected to chromatin immunoprecipitation analysis using a ChIP kit, following the manufacturer's instructions. Parallel immunoprecipitation, using rabbit IgG, was performed as a negative control. Chromatin-immunoprecipitated DNA was analyzed by RT-qPCR. The genetic regions of the FBP1 gene loci for VDR binding were analyzed using the JASPAR website (https://jaspar.genereg.net/) and two potential regions were predicted. Primers targeting the promoter region within of FBP1 were used. Primer pair sequences are listed in Table S3.

### Dual-Luciferase Reporter Assay

HEK293T cells were seeded in a 24-well plate and transfected with the indicated plasmids (PGL4.10 control vector, wild-type FBP1-promoter, FBP1-promoter mutation-site 1, and FBP1-promoter mutation-site 2 from TransSheep Biotechnology) for 24 h followed by 1α,25(OH)_2_D_3_ activation for another 12 h. FLuc/RLuc activity was measured using the Dual Luciferase Reporter Assay System (MCE).

### Flow cytometry

For cell surface staining, PBMCs or SVF were incubated with specific antibodies for 20 min at 4°C in the dark. For intracellular cytokine staining, cells were stimulated with 50 ng/mL phorbol 12-myristate 13-acetate (PMA) and 1 μg/mL ionomycin (Ion) in the presence of Golgi Stop for 4 h*.* The cells were then stained with surface markers, fixed, and permeabilized with BD Cytofix/Cytoperm™ Plus. Samples were acquired on BD Verse/Cytek Biosciences, and data were analyzed using FlowJo v. 10 (FlowJo LLC) software. For the FBP1 inhibition experiment, 1α,25(OH)_2_D_3_ pretreated Vδ2 T cells were re-stimulated with IL-2 and 1α,25(OH)_2_D_3_ for 20 h, with or without MB05032 (200 μM) followed by PMA+Ion activation for another 4 h. In some experiments, 10 μM of SB203580 was added. Cell surface and intracellular cytokine staining was performed as described previously. For proliferation detection, Vδ2-T cells were preloaded with 2.5 μM CFSE. The cells were then treated with vehicle, 1α,25(OH)_2_D_3_, or 1α,25(OH)_2_D_3_ combined with MB05032 for 48 h, followed by flow cytometry. The antibodies used are listed in Table S4.

### Metabolism measurements

The extracellular acidification rate (ECAR) and oxygen consumption rate (OCR) were determined using an XF96 Extracellular Flux Analyzer. 1α,25(OH)_2_D_3_ pretreated Vδ2 T cells were re-stimulated with vehicle or 100 nM 1α,25(OH)_2_D_3_ for 24 h, with or without 200 μM MB05032. Vδ2 T cells were then collected and resuspended in XF Base Medium (pH 7.4) with L-glutamine (2 mM) and were then placed into a cell culture microplate (1.5×10^5^ cells per well). For the ECAR tests, glucose (10 mM), oligomycin (1 μM), and 2-DG (50 mM) were added to the cells at the indicated times. The reagents included XF Base medium containing 10 mM glucose, 1 μM oligomycin and 50 mM 2-DG (ECARs), or 2 mM glutamine, 1 mM pyruvate, 10 mM glucose, 1 μM oligomycin, 0.25 μM FCCP, 0.5 μM rotenone, and antimycin A (OCRs), all procedures were following the manufacturer's recommendations.

### Constructs

The human VDR gene was amplified from the Vδ2-T cell cDNA library. sgRNAs for VDR knockout were inserted into a lenti-CRISPR v.2 vector with the puromycin gene as a screening marker. The sgRNA sequence for human VDR knockout was 5'-GATGCGGCAGTCCCCGTTGA-3'. Packaging plasmids for the lentiviruses psPAX2 and PMD.2G were purchased from Addgene.

### Generation of VDR knockout Vδ2 T cell

For VDR knockout, HEK293T cells were transfected with packaging plasmids (PMD.2G and psPAX2) and lenti-CRISPR v.2-based or knockout vectors using Lipofectamine 3000, according to the manufacturer's instructions. Virus-containing supernatants were collected at 60 h after transfection and concentrated using a Lenti-X Concentrator. For lentivirus transduction, Vδ2 T cells were incubated with the lentivirus at 500×g for 90 min at 4 ℃. Subsequently, the cells were cultured in a normal medium for an additional 72 h. After puromycin selection three times, T cells were detected using qPCR or blotting. In some experiments, transduced T cells were re-stimulated with 100 nM 1α,25(OH)_2_D_3_ three times at 1-day intervals and harvested for further analysis.

### Western blot analysis

1α,25(OH)_2_D_3_ pretreated Vδ2 T cells were re-stimulated with 1α,25(OH)_2_D_3_ (100 nM) for 6, 10, and 24 h, with or without MB05032 (200 μM). Cells were then lysed in RIPA buffer containing protease inhibitor and phosphatase inhibitor cocktails on ice for 30 min, and the supernatants were used for subsequent analysis. Proteins were then transferred to 0.45 μm polyvinylidene fluoride (PVDF) membranes, and blocked with 5% bovine serum albumin (BSA) for 2 h at room temperature. Membranes were incubated with primary antibodies overnight at 4 ℃. After washing six times in TBST for 1 h, the membranes were incubated with HRP-conjugated secondary antibodies for 2 h at room temperature, and finally detected using the Bio-Rad ChemiDoc MP Gel imaging system.

### Immunofluorescence

The cells were fixed in 4% paraformaldehyde for 20 min. After blocking with BSA (2%), the cells were permeabilized with 0.2% Triton X-100 for 25 min and blocked in Tris-buffered saline (0.02% Triton X-100 and 2% BSA in PBS) for 2 h. The cells were then stained with secondary antibody for another 2 h. All the images were captured using a confocal microscope.

### Glucose uptake

Briefly, Vδ2 T cells (7.5×10^5^/mL) pretreated with 1α,25(OH)_2_D_3_ or vehicle were cultured in fresh medium with or without MB05032 (200 μM) in the presence of glucose (2 mM) for 0, 24, 48, and 72 h. Glucose levels in the culture supernatants were measured using a glucose (GO) Assay Kit following the manufacturer's instructions.

### ELISA

Human serum samples were collected as previously described 43. Assays using ELISA kits for human and mouse 25-hydroxyvitamin D_3_ and TNF-α (Jianglai Biotech) were performed according to the manufacturer's instructions.

### Serum biochemistry

Whole blood samples of T2D patients were collected, and sera were analyzed using an automatic biochemistry analyser (7600-020, Hitachi).

### RNA sequencing

Transcriptome sequencing and analysis were conducted using BGI technology. KEGG pathway enrichment analysis of DEGs was performed using the phyper function in the R software. Rich Ratio=Term Candidate Gene Number/Term Gene Number. For volcano plot analysis, the X-axis was defined as fold change (FC)=log2(1.25D_3_/vehicle), and the Y-axis was calculated as -log10 (Q value). Chord diagram analysis for part of DEGs (FC≤-2 or FC≥2) was performed using https://www.bioinformatics.com.cn, a free online platform for data analysis and visualization. For expression cluster analysis, gene expression levels were normalized to log2 (TPM+1). To investigate the biological states or functional differences of Vδ2 T cells under the condition of 1α,25(OH)_2_D_3_ or vehicle treatment, the DEGs between treated/control cells were used to investigate hallmark gene sets by Gene Set Enrichment Analysis (GSEA).

### Statistical analysis

Statistical analyses and graphs were performed using GraphPad Prism (v.9). Data were obtained from biologically independent samples. Data are presented as the mean ± standard deviation (SD).

## Results

### Vitamin D deficiency aggravates inflammatory responses of γδ T cells in patients with type 2 diabetes

**Figure 1:**
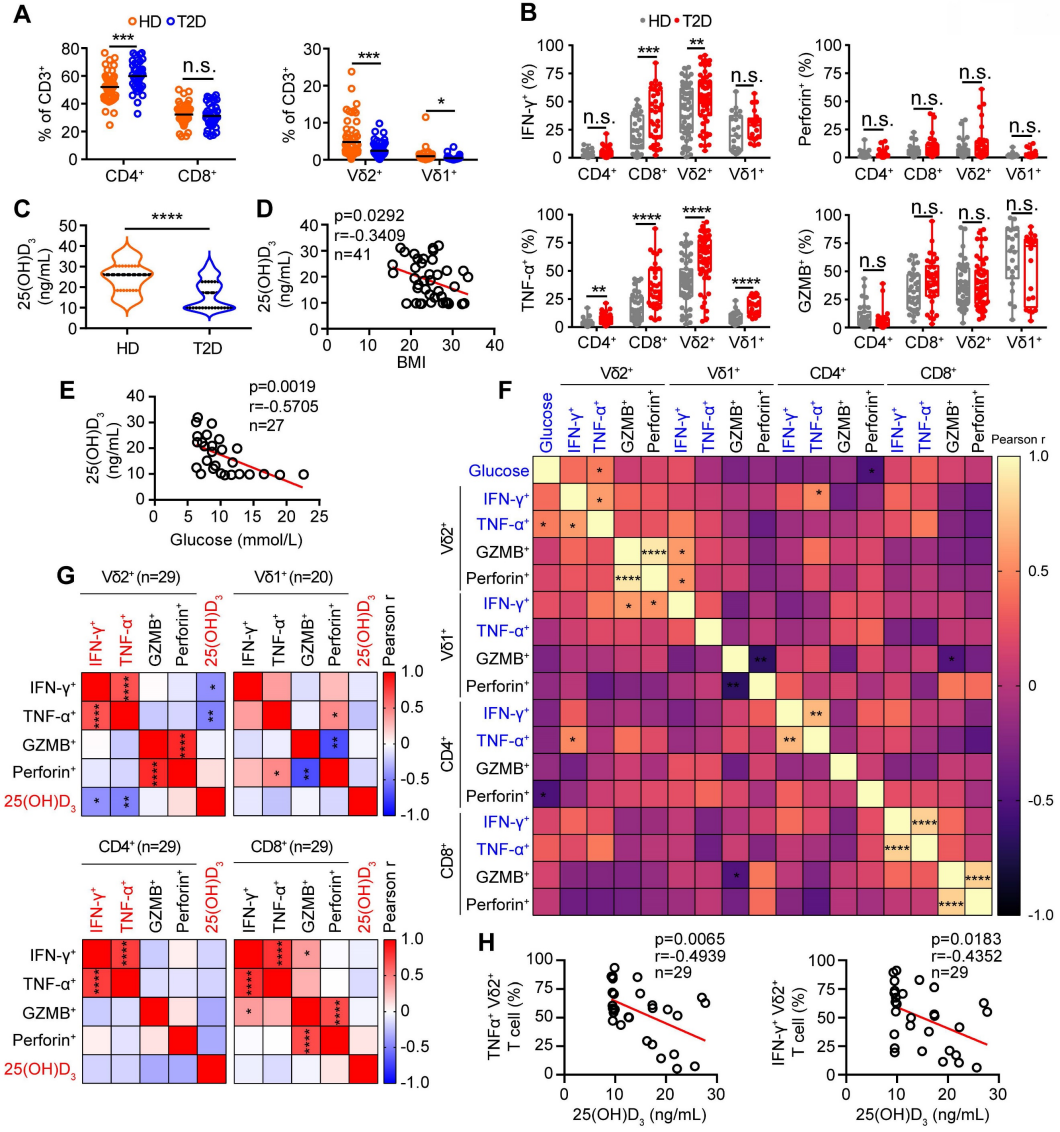
** Vitamin D deficiency aggravates inflammatory responses of γδ T cells in patients with type 2 diabetes. (A** and** B)** The percentages of CD4, CD8 (T2D, n = 36; HD, n = 52), Vδ1, and Vδ2 (T2D, n = 56; HD, n = 66) out of CD3^+^ T cells, and the cytokine production of T cells from HD (healthy donors) and T2D were analyzed by FACS (T2D, n = 20-55; HD, n = 22-56). **(C)** Serum 25(OH)D_3_ levels in T2D and HD were detected by ELISA (HD, n = 25; T2D, n = 68). **(D)** The correlation of BMI and serum 25(OH)D_3_ level in T2D was analyzed. **(E)** Correlation between fasting blood glucose and 25(OH)D_3_ levels in T2D patients. **(F)** Linear regression analysis of FBG levels and cytokines in T cells (CD4, CD8, Vδ1, and Vδ2; T2D, n = 17). **(G** and** H)** Correlation between serum 25(OH)D_3_ levels and cytokines in CD4, CD8, Vδ1, and Vδ2 T cells in T2D. Statistical analysis was performed using two-tailed unpaired Student's *t*-tests (A; B, IFN-γ^+^ Vδ2^+^, TNF-α^+^ Vδ1^+^, GZMB^+^ CD8^+^); Mann-Whitney test (B, Vδ2^+^/Vδ1^+^ CD3^+^, IFN-γ^+^ CD4^+^/CD8^+^/Vδ1^+^, TNF-α^+^ CD4^+^/CD8^+^/Vδ2^+^, GZMB^+^ CD4^+^/Vδ2^+^/Vδ1^+^, Perforin^+^ CD4^+^/CD8^+^/Vδ2^+^/Vδ1^+^; C); Pearson's correlations (D-H). Data represent mean ± SD. *P < 0.05, **P < 0.01, ***P < 0.001, ****P < 0.0001. n.s., not significant.

Low-serum vitamin D level was correlated with increased risk of chronic inflammation, insulin resistance and T2D 1, 31. Therefore, to investigate the role of vitamin D in the function of γδ T cells, we recruited T2D patients to detect the percentage and cytokine secretion of these cells, and a summary of their characteristics is provided in Table S1. We found that the percentage of circulating Vγ9Vδ2 (Vδ2) T cells in T2D patients was significantly lower than that of healthy donors (**Figure 1A**). Importantly, the expression of cytokine-related genes in circulating γδ T cells was detected by quantitative real-time PCR, and the results showed that the levels of TNF-α and IFN-γ were increased in T2D patients (**Figure S1A**). We also observed that the production of circulating TNF-α^+^ CD4^+^, TNF-α^+^ CD8^+^, TNF-α^+^ Vδ2^+^, TNF-α^+^ Vδ1^+^, IFN-γ^+^ CD8^+^, and IFN-γ^+^ Vδ2^+^ T cells was significantly increased compared to that in healthy donors, while granzyme B and perforin levels were not significantly changed in T2D (**Figure 1B**, **Figure S1B-D**). We next examined the serum level of 25(OH)D_3_ using an enzyme-linked immunosorbent assay (ELISA) and found that it was indeed decreased in T2D patients (**Figure 1C**). The serum 25(OH)D_3_ level in T2D patients was negatively correlated with BMI and fasting blood glucose (FBG) (**Figure 1D-E**). Furthermore, the levels of TNF-α and IFN-γ in circulating Vδ2 T cells were positively correlated with FBG levels in T2D individuals (**Figure 1F**). Interestingly, the expression of TNF-α and IFN-γ in circulating Vδ2 T cells further increased as the degree of obesity increased, and was also inversely correlated with serum 25(OH)D_3_ in T2D (**Figure S2A-C**, **Figure 1G-H**). These results suggest that vitamin D may be involved in the development of obesity, γδ T-cell activation, and insulin resistance.

### 1α,25(OH)_2_D_3_ alleviates inflammatory responses of γδ T cells and insulin resistance in obese mice

**Figure 2:**
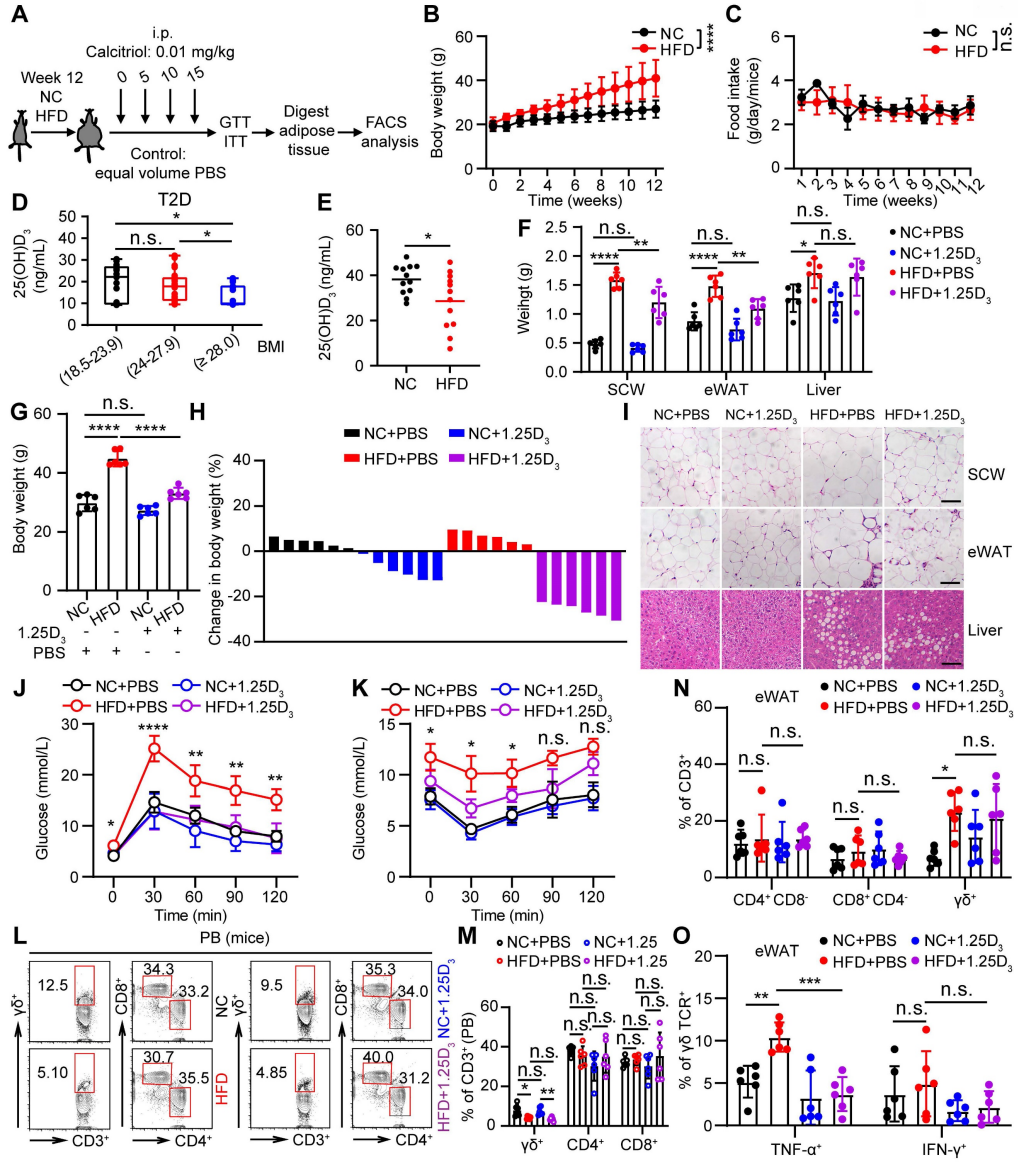
** 1α,25(OH)_2_D_3_ alleviates inflammatory responses of γδ T cells and insulin resistance in obese mice. (A)** Overview of experimental design. **(B)** WT mice were fed high-fat diet (HFD) or normal chow (NC) for 12 weeks (n = 12 per group). Body weight was recorded weekly. **(C)** Daily food intake of the mice (n = 3 per group). **(D)** Serum 25(OH)D_3_ levels in T2D patients. BMI (kg/m^2^) was categorized as healthy weight (18.5-23.9, n = 27), overweight (24.0-27.9, n = 25), and obesity (≥ 28.0, n = 16). **(E)** Serum 25(OH)D_3_ levels in mice were measured using ELISA after NC and HFD for 12 weeks (n = 12 per group). **(F-I)** Mice were fed a HFD for 12 weeks and then intraperitoneally injected with rocaltrol (1α,25(OH)_2_D_3_) or PBS. Tissues (SCW, eWAT, and liver) were weighed (F, n = 6), and body weight was recorded at the indicated time points (G, n = 6). Waterfall plot of the percentage of mice showing weight change after treatment with rocaltrol or PBS (H, n = 6). Hematoxylin and eosin (H&E) staining of the tissues is shown (I). Scale bars, 50 μm. **(J-K)** GTT and ITT measurements were performed after rocaltrol treatment for 15 days (n = 6, HFD+PBS vs HFD+1.25D_3_). **(L-M)** Mouse PBMCs (PB) were analyzed by FACS after feeding with HFD, NC, HFD+1.25D_3_ or NC+1.25D_3_ for 12 weeks (n = 6). **(N-O)** The percentage of CD4, CD8, and γδ T cells infiltrated into adipose tissues, and the cytokine production of γδ T cells from eWAT was analyzed by FACS (n = 6). Two-tailed unpaired Student's *t*-tests (E), one-way ANOVA with Tukey's multiple comparisons test (D, F, G, M, N, and O), two-way ANOVA (B-C), and two-way ANOVA with Tukey's multiple-comparisons test (J-K). Data are represented as the mean ± SD. *P < 0.05, **P < 0.01, ***P < 0.001, ****P < 0.0001. n.s., not significant.

To understand the role of vitamin D in regulating obesity, inflammation, and insulin resistance, we fed C57BL/6J wild-type (WT) mice a high-fat diet (HFD) and normal chow (NC) as controls (**Figure 2A**). The body weights of obese mice were significantly higher than those of NC mice, while there were no significant differences in food intake between NC and HFD mice (**Figure 2B-C**). Importantly, serum levels of 25(OH)D_3_ were significantly decreased in both T2D and obese mice (**Figure 2D-E**). Therefore, we tested whether vitamin D has the potential to treat obesity and insulin resistance. We treated obese WT mice with 1α,25(OH)_2_D_3_ and found that the body weights of obese mice and their adipose size were reduced (**Figure 2F-I**), but without a change in food intake (**Figure S3A**). In addition, 1α,25(OH)_2_D_3_ administration substantially ameliorated glucose intolerance and insulin resistance in HFD-induced obese mice (**Figure 2J-K**). Consistent with the data from T2D patients, the percentage of peripheral circulating γδ T cells was significantly decreased in HFD-induced obese mice, whereas the percentage of γδ T cells was barely affected by 1α,25(OH)_2_D_3_ treatment (**Figure 2L-M**). Visceral adipose tissue (VAT) is a more pathogenic depot than subcutaneous white adipose tissue (SCW), and an increased VAT correlates with a high risk of metabolic syndrome and T2D 44, 45. We also found that HFD-fed mice developed enhanced inflammatory responses in epididymal white adipose tissue (eWAT), as indicated by increased γδ T cell infiltration and higher inflammatory cytokine production, while TNF-α levels were reversed by 1α,25(OH)_2_D_3_ treatment *in vivo* (**Figure 2N-O**, **Figure S3B-E**). Furthermore, we found that the IL-17A levels both in circulating total γδ T cells and adipose tissue residual γδ (Vγ1 and Vγ4) T cells in obese mice were higher than those in normal chow mice, whereas the level of IL-17A was barely affected by 1α,25(OH)_2_D_3_ (**Figure S4A-D**). Importantly, the levels of IFN-γ^+^ and TNF-α^+^, both in adipose tissue residual Vγ1/Vγ4 T and circulating γδ T cells, were reduced by 1α,25(OH)_2_D_3_ treatment (**Figure S4E-G**). Collectively, these results demonstrate that vitamin D has a protective effect in against obesity and reduces the production of inflammatory cytokines.

### 1α,25(OH)_2_D_3_ induces the expression of FBP1 in Vδ2 T cells

**Figure 3:**
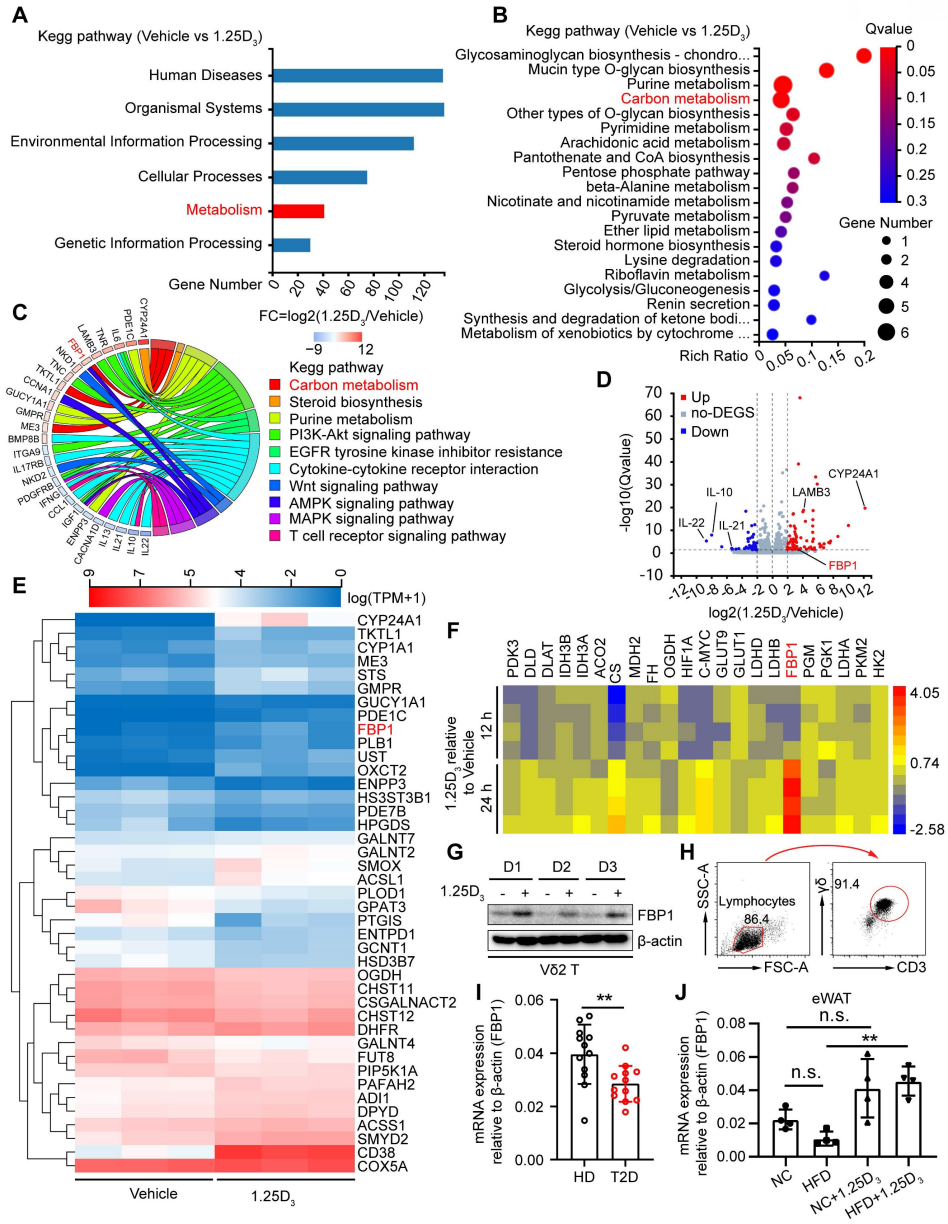
** 1α,25(OH)_2_D_3_ induces the expression of FBP1 in Vδ2 T cells. (A)** 1α,25(OH)_2_D_3_- or vehicle-pretreated Vδ2 T cells were collected for RNA-seq analysis. Differentially expressed genes (DEGs) were analyzed using KEGG pathway enrichment. **(B)** Total 41 differentially expressed genes from the metabolic pathway (Fig. 3A) were used for KEGG pathway enrichment analysis.** (C)** Chord plot showing partial DEGs enriched in the pathways. **(D)** Volcano plot showing the significantly regulated genes in Vδ2 T cells after 1α,25(OH)_2_D_3_ treatment. **(E)** Vδ2 T cells were treated with 1α,25(OH)_2_D_3_ three times and cells were collected for RNA-seq analysis (n = 3). **(F-G)** FBP1 expression was detected using qPCR (F, n = 4) and immunoblotting (G, n = 3). **(H)** Circulating γδ T cells were isolated from patients with T2D by EasySep Human Gamma/Delta T cell Isolation Kit. The purity of the total γδ T cells was determined using FACS. **(I)** Relative mRNA levels of FBP1 in circulating γδ T cells derived from PBMCs of HD and T2D patients (HD, n = 12; T2D, n = 12). **(J)** Relative mRNA expression of FBP1 in eWAT derived from HFD-fed or normal chow-fed mice (n = 5). Two-tailed unpaired Student's *t*-test (I); one-way ANOVA with Tukey's multiple comparisons test (J). Data are represented as the mean ± SD. *P < 0.05, **P < 0.01, ***P < 0.001, ****P < 0.0001. n.s., not significant.

To investigate the molecular mechanism by which vitamin D regulates metabolic reprogramming, we performed transcriptomic analysis of human Vδ2 T cells treated with 1α,25(OH)_2_D_3_ *in vitro*. Differentially expressed genes (DEGs) were identified and subjected to KEGG analysis, and 41 DEGs in the metabolic pathway were further analyzed by KEGG pathway enrichment (**Figure 3A**-**B**). The expression of FBP1 was substantially induced after 1α,25(OH)_2_D_3_ treatment, and the key genes were also enriched in metabolism- related signaling pathways (**Figure 3C-E**). Indeed, 1α,25(OH)_2_D_3_ up-regulated expression of FBP1, which was further confirmed by quantitative RT-PCR and immunoblotting (**Figure 3F-G**). Interestingly, reduced expression of FBP1 in circulating γδ T cells was detected in patients with T2D (**Figure 3H-I**). Furthermore, we collected adipose tissue samples from HFD- and normal chow- fed mice. Quantitative Real-time PCR showed that the level of FBP1 in eWAT was reduced in HFD mice compared that in normal chow, whereas the expression of FBP1 was increased by 1α,25(OH)_2_D_3_ treatment (**Figure 3J**). These data show that 1α,25(OH)_2_D_3_ enhanced the expression of FBP1 in activated γδ T cells.

### 1α,25(OH)_2_D_3_/VDR signaling promotes FBP1 activity to restrain glycolysis

**Figure 4:**
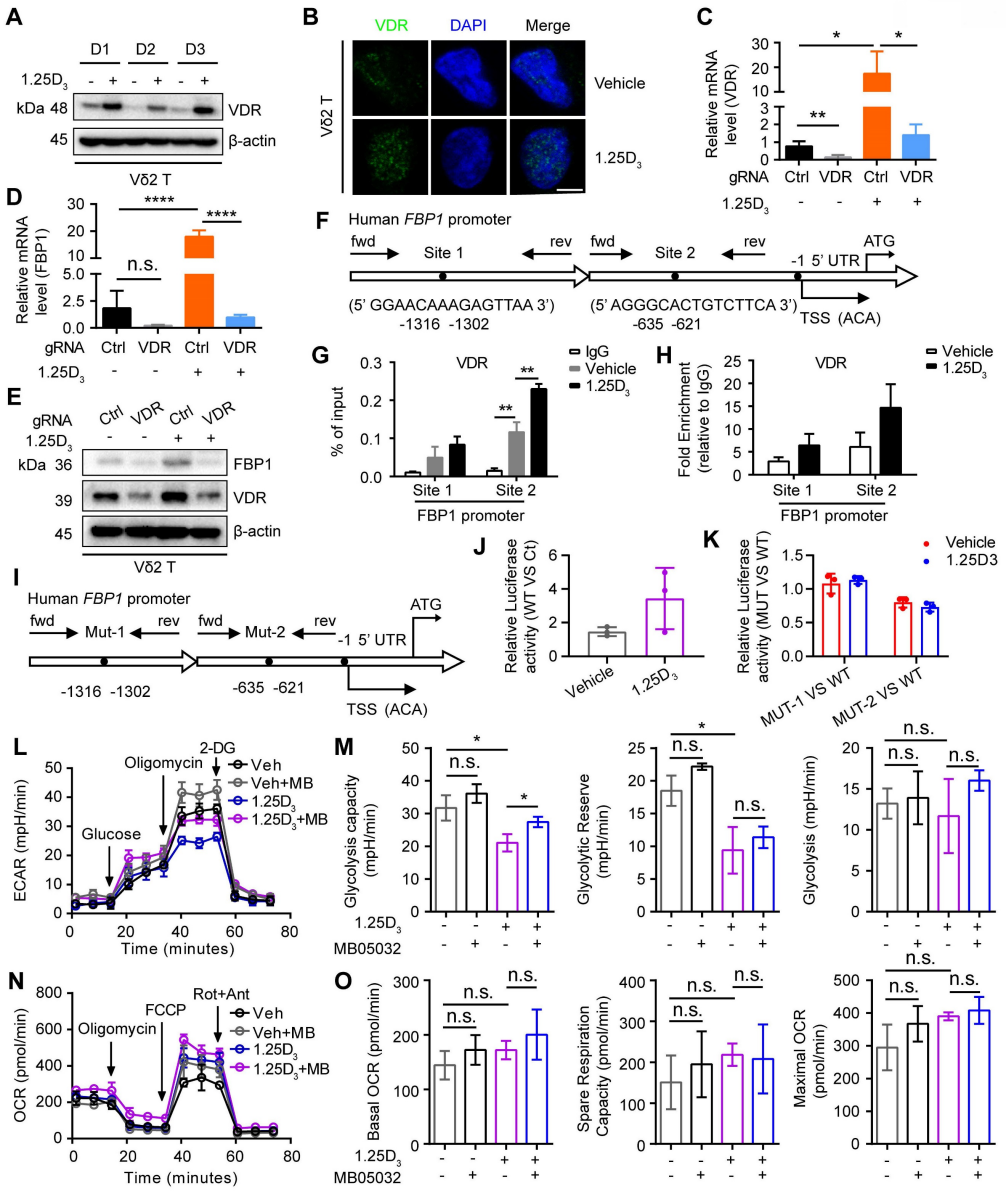
** 1α,25(OH)_2_D_3_/VDR signaling promotes FBP1 activity to restrain glycolysis. (A** and** B)** Expression of VDR in 1α,25(OH)_2_D_3_-treated Vδ2 T cells was detected by immunoblotting (A, n = 3) and immunofluorescence (B). **(C-E)** Vδ2 T cells were transfected with VDR knockout vector (gRNA target VDR) or control vector (Ctrl) and then treated with 1α,25(OH)_2_D_3_ three times at 1-day intervals. The levels of target genes and proteins were detected by qPCR (n = 4) and immunoblotting, respectively. **(F-H)** Predicted VDR-binding sites in the human FBP1 promoter region (F). ChIP assay for two potential VDR binding sites within the FBP1 promoter was performed with anti-VDR in 1α,25(OH)_2_D_3_-treated Vδ2 T cells (G-H, n = 3).** (I)** Promoter mutations in FBP1 gene. **(J-K)** The dual-luciferase reporter system detects the relative binding activity of VDR to the FBP1 promoter. WT (FBP1 promoter), Ct (control vector), Mut-1 (FBP1-promoter mutation site 1), and Mut-2 (FBP1-promoter mutation site 2). **(L-O)** Vδ2 T cells were collected to detect ECAR (L-M) and OCR (N-O) using an extracellular flux analyzer. Cumulative data for the calculated glycolysis capacity, glycolytic reserve, glycolysis, basal OCR, spare respiration capacity, and maximal OCR are shown (n = 6). One-way ANOVA with Tukey's multiple comparisons test (C, D, G, M, and O). Data are represented as the mean ± SD. *P < 0.05, **P < 0.01, ***P < 0.001, ****P < 0.0001. n.s., not significant.

Next, we determined whether 1α,25(OH)_2_D_3_ promoted FBP1 expression in γδ T cells through the vitamin D receptor (VDR) signaling pathway and found that 1α,25(OH)_2_D_3_ increased VDR expression and induced its nuclear translocation in Vδ2 T cells (**Figure 4A-B**). In order to further confirm the role of VDR on FBP1 expression, we generated VDR-knockout Vδ2 T cells and the results showed that the expression of FBP1 in VDR-knockout Vδ2 T cells was dampened even under the treatment of 1α,25(OH)_2_D_3,_ while the expression of VDR and FBP1 was enhanced after 1α,25(OH)_2_D_3_ stimulation in control groups (**Figure 4C-E**). 1α,25(OH)_2_D_3_ binds to VDR, triggers its nuclear translocation, and regulates the transcription of multiple genes 46, 47. Therefore, VDR may act as a transcription factor for the expression of FBP1. To test this hypothesis, we used the JASPAR website to predict the VDR binding sites in the promoter region of *FBP1* (**Figure 4F**). ChIP-qPCR analysis showed that VDR levels in the *FBP1* promoter were also notably increased (**Figure 4G-H**). Furthermore, the control plasmid, wild-type FBP1-promoter, and FBP1-promoter mutation-site 1/2 were constructed the PGL4.10 vector. The dual-luciferase reporter assay showed that VDR mainly binds at predicted site 2 to promote FBP1 expression (**Figure 4I-K**). To determine whether the effect of 1α,25(OH)_2_D_3_ treatment on γδ T cells is attributed to decreased metabolism, we next analyzed glycolysis and aerobic respiration by seahorse. Interestingly, inhibition of Extracellular Acidification Rate (ECAR), such as glycolysis capacity in response to 1α,25(OH)_2_D_3_, was significantly reversed by the FBP1 inhibitor (MB05032), while the Oxygen Consumption Rate (OCR), including basal OCR, spare respiration, and maximal OCR barely changed (**Figure 4L-O**, **Figure S5A**). Meanwhile, the proliferation and apoptosis of Vδ2 T cells was not significantly inhibited by 1α,25(OH)_2_D_3_, MB05032 or their combination treatment in a short time (**Figure S5B-D**). Taken together, these results demonstrate that the 1α,25(OH)_2_D_3_/VDR signaling pathway promotes FBP1 expression to restrain glycolysis in γδ T cells.

### 1α,25(OH)_2_D_3_ restrains cytokine production in Vδ2 T cells through the FBP1/Akt/p38 MAPK pathway

**Figure 5:**
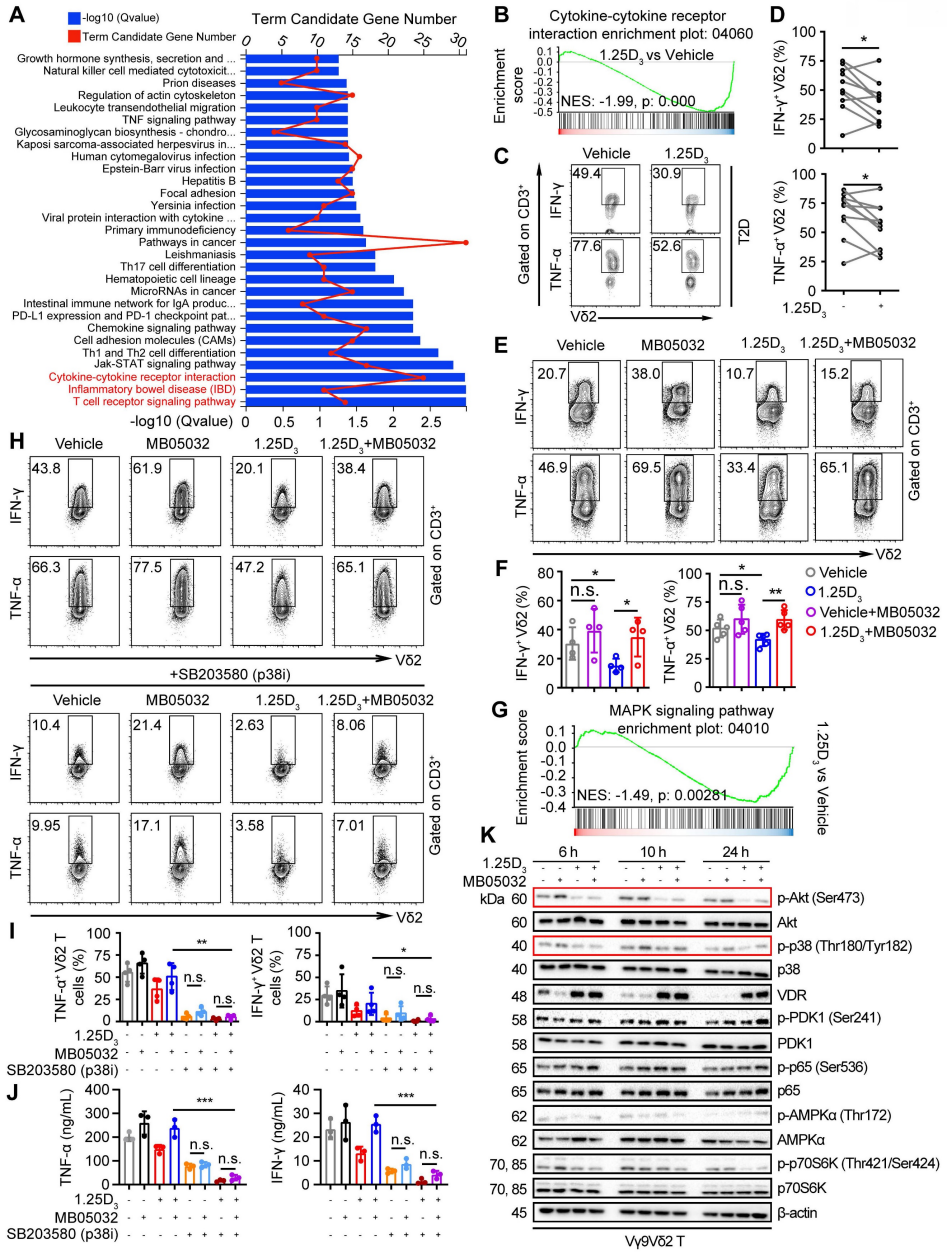
** 1α,25(OH)_2_D_3_ restrains cytokine production in Vδ2 T cells through the FBP1/Akt/p38 MAPK pathway. (A)** Bar chart showing the enrichment of specific pathways based on DEGs of vehicle- or 1α,25(OH)_2_D_3_-treated Vδ2 T cells. **(B)** GSEA was performed for indicated pathways. NES, normalized enrichment score. **(C** and** D)** FACS and statistical analysis of cytokines in Vδ2 T cells from patients with T2D after 1α,25(OH)_2_D_3_ or vehicle treatment *in vitro* (n = 10). (**E** and** F**) Vδ2 T cells were treated with 1α,25(OH)_2_D_3_ or vehicle, followed by re-stimulation with 1α,25(OH)_2_D_3_, MB05032, or their combination for another 20 h. FACS and statistical analysis of the percentage of cytokines are shown (n = 4-5). **(G)** GSEA analysis was performed for MAPK signaling pathway. **(H-J)** Vδ2 T cells were treated with 1α,25(OH)_2_D_3_ and MB05032 for 20 h, followed by SB203580 treatment or no treatment for another 4 h. The percentage of cytokine production was analyzed using FACS and statistical analysis (n = 4). Serum cytokine levels were determined using ELISA (n = 3). **(K)** 1α,25(OH)_2_D_3_ pretreated Vδ2 T cells were restimulated with 1α,25(OH)_2_D_3_, MB05032, or their combination for 6, 10, and 24 h, and then harvested for immunoblot analysis. Paired Student's t-test (D); one-way ANOVA with Tukey's multiple comparisons test (F, I and J). Data are represented as the mean ± SD. *P < 0.05, **P < 0.01, ***P < 0.001, ****P < 0.0001. n.s., not significant.

Glycolysis is specifically required for effector cytokine production in T-cells 34, 35, 48. KEGG pathway and gene set enrichment analyses (GSEA) revealed that 1α,25(OH)_2_D_3_ treatment resulted in alterations in multiple pathways, including the cytokine-cytokine receptor interaction pathway (**Figure 5A-B**). Reduced expression of several proinflammatory factor genes was detected in 1α,25(OH)_2_D_3_ pretreated Vδ2 T cells (**Figure S6A-B**). Thus, we collected peripheral blood mononuclear cells (PBMCs) from T2D patients to detect cytokine production. Indeed, 1α,25(OH)_2_D_3_ pretreated PBMCs *in vitro* showed diminished production of IFN-γ and TNF-α in Vδ2^+^ CD3^+^ T cells (**Figure 5C-D**). Previous results showed that 1α,25(OH)_2_D_3_ mediated FBP1 expression in γδ T cells was associated with multiple metabolic pathways (Figure 3c); therefore, we speculated that FBP1 might participate in the regulation of cytokine production by γδ T cells. We found that the inhibition of cytokine production in response to 1α,25(OH)_2_D_3_ was significantly restored by MB05032 treatment, whereas perforin and granzyme B in these cells remained unchanged upon activation with phorbol 12-myristate 13-acetate and ionomycin (**Figure 5E-F**, **Figure S6C-D**). To further investigate the molecular mechanism underlying the impairment of cytokine production after 1α,25(OH)_2_D_3_ stimulation, we performed GSEA, and the results showed that 1α,25(OH)_2_D_3_ modulated the expression of genes involved the MAPK signaling pathway (**Figure 5G**). More importantly, upregulation of TNF-α and IFN-γ with MB05032 treatment was significantly reduced in response to p38 MAPK inhibition (SB203580) in 1α,25(OH)_2_D_3_ treated Vδ2 T cells (**Figure 5H-J**). Meanwhile, the phosphorylation of Akt and p38 MAPK was significantly decreased after restimulation with 1α,25(OH)_2_D_3_, while it was exact opposite after treatment with the FBP1 inhibitor (**Figure 5K, Figure S6E**). Collectively, these findings indicate that 1α,25(OH)_2_D_3_ represses cytokine production through the FBP1/Akt/p38 MAPK pathway in γδ T cells.

### 1α,25(OH)_2_D_3_ mediates the activity of FBP1 in γδ T cells to alleviate insulin resistance in obese mice

**Figure 6:**
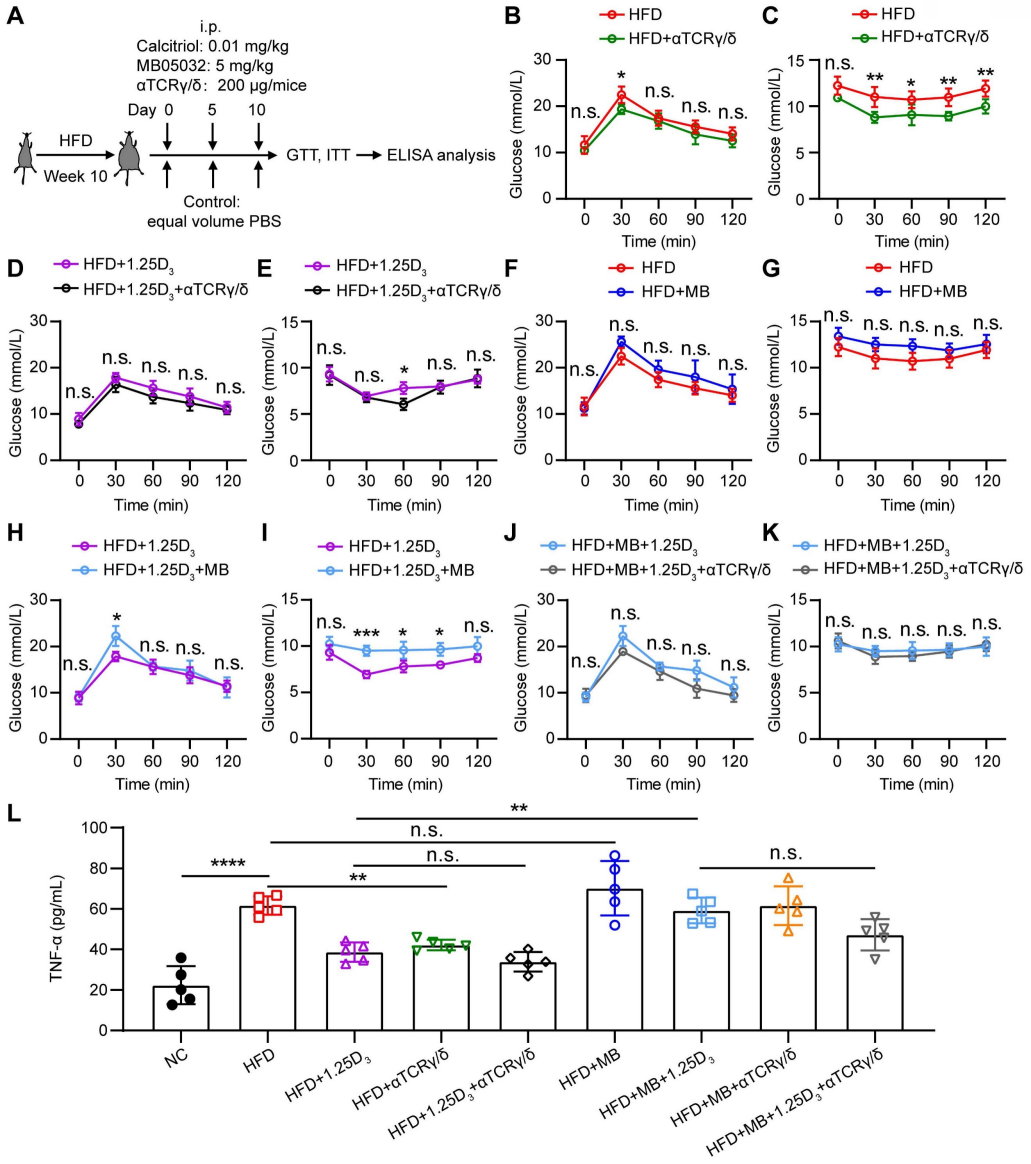
** 1α,25(OH)_2_D_3_ mediates the activity of FBP1 in γδ T cells to alleviate insulin resistance in obese mice. (A)** Overview of study design. WT mice were fed a high-fat diet (HFD) or normal chow (NC) for 10 weeks (n = 5 per group). **(B-K)** GTT and ITT in mice after treatment with PBS, rocaltrol (1.25D_3_), MB05032 (MB), and anti-TCRγ/δ (αTCRγ/δ) alone or in combination for 10 days. **(L)** Serum TNF-α levels in NC and HFD mice were measured by ELISA after treatment with PBS, rocaltrol, MB05032, anti-TCRγ/δ alone, or their combination for 10 days (n = 5 per group). Data are represented as mean ± SD; Two-way ANOVA with Tukey's multiple-comparisons test (B-K); one-way ANOVA with Tukey's multiple comparisons test (L); *P < 0.05, **P < 0.01, ***P < 0.001, ****P < 0.0001. n.s., not significant.

To determine whether 1α,25(OH)_2_D_3_/FBP1 signaling could alleviate insulin resistance through γδ T cells, HFD mice were treated with anti-mouse TCRγ/δ antibody, FBP1 inhibitor MB05032, 1α,25(OH)_2_D_3_, alone or in combination (**Figure 6A**). We found that depletion of γδ^+^ T cells significantly reduced insulin resistance in HFD mice (**Figure 6B-E**), suggesting that γδ T cells may contribute to insulin resistance. However, in the HFD mouse model, inhibition activity of FBP1 marginally induced glucose levels compared with the vehicle group (**Figure 6F-G**). To further investigate whether 1α,25(OH)_2_D_3_/FBP1 signaling could reverse insulin resistance by directly modulating γδ T cells, HFD mice were treated with anti-mouse TCRγ/δ antibody to deplete γδ^+^ T cells under MB05032 and 1α,25(OH)_2_D_3_ treatment. Interestingly, depletion of γδ^+^ T cells was only marginally decreased glucose levels compared to the combination of MB05032 and 1α,25(OH)_2_D_3_ treatment (**Figure 6H-K**). Importantly, decreased serum TNF-α production during the depletion of γδ^+^ T cells was also observed in HFD mice (**Figure 6L**). Taken together, our data indicated that 1α,25(OH)_2_D_3_/FBP1 signaling could regulate the inflammation of γδ T cells to alleviate insulin resistance in obese mice.

## Discussion

Increasing evidence indicates that many of the comorbidities of obesity, including T2D, nonalcoholic fatty liver, and cancer, are related to chronic inflammation 26, 27. However, the mechanisms that of trigger this inflammation are not yet fully understood. Reilly et al. summarized several potential mechanisms, including the triggering of adipose tissue inflammation that could emanate from gut-derived substances, dietary components, or metabolites 27. From an immunological point of view, adipose tissue-infiltrating immune cells (αβ, γδ T, NK, and macrophages), which initiate inflammation in obese adipose tissue, contribute to insulin resistance and the progression of T2D 18, 21-24, 27, 49. We observed that inflammatory hallmarks such as IFN-γ and TNF-α in γδ T cells were increased both in T2D related obesity or HFD-induced obesity mice using PMA and Ionomycin stimulation, suggesting that obesity impaired γδ T cell homeostasis, which is related to inflammatory cytokine secretion.

Donath et al. discussed the rationale and effect of some of anti-inflammatory treatments in patients with diabetes, including IL-1 receptor blockade, IL-1β antagonism, and TNF antagonism, which showed protective effects in T2D 50. Several challenges remain in the development of anti-inflammatory drugs for the treatment of T2D, such as side effects, drug safety and cost of treatment. Interestingly, we found that the serum level of 25(OH)D_3_ in T2D was significantly reduced as BMI increased, and it was negatively correlated with the levels of TNF-α and IFN-γ from circulating γδ T cells, indicating the therapeutic potential of vitamin 25(OH)D_3_ in controlling T cell inflammatory responses. γδ T cells resided significantly in obese adipose tissue in HFD-induced obese mouse models. Notably, therapeutic administration of vitamin 1α,25(OH)_2_D_3_ decreased inflammatory cytokine secretion by γδ T cells and substantially ameliorated insulin resistance in obese mice. However, our recent study demonstrated that 1α,25(OH)_2_D_3_ pretreated γδ T cells, activated by anti-CD3/CD28 antibody or tumor cells, showed increased Th1 cytokine production 43. Bernicke et al. found seasonal fluctuations in γδ T cells and immunomodulatory effects of vitamin D 51. Further investigation is required to explain the pleiotropic role of vitamin D in different diseases.

Much attention has been focused on the regulation of the catabolic pathway of glucose, and gluconeogenesis is less investigated and may play an equally important role in the switch to glycolysis in tumor cells and natural killer cells 39, 52. Hunter et al. report a new mechanism of action for metformin and provide further evidence that molecular targeting of FBP1 can have antihyperglycemic effects 53. RAN-seq data also showed that vitamin D treatment promoted the expression of multiple genes in human Vδ2 T cells, including CYP24A1, SMOX, ACSL1, and CD38. However, whether the expression of these genes is mediated by vitamin 1α,25(OH)_2_D_3_ and its influences on γδ T cell metabolism and proinflammatory cytokine production has not been investigated.

Our work provides evidence that 1α,25(OH)_2_D_3_/VDR/FBP1 signaling suppresses cytokine production by γδ T cells via inhibition of glycolysis and phosphorylation of the Akt/p38 MAPK pathway. T2D is a complex disease, and ZOL-expanded γδ T cells from healthy donors after 1α,25(OH)_2_D_3_ treatment for RNA-seq studies may not fully mimic γδ T cells residing in the adipose tissues of obese mice or T2D patients. Moreover, our results showed that administration of 1α,25(OH)_2_D_3_ reduced body weight and the level of inflammatory cytokines in circulating and tissue residual γδ T cells. Khosravi et al. reported that vitamin D supplementation for 6 weeks, the BMI was decreased significantly compared to control group 54. However, the mechanism by which 1α,25(OH)_2_D_3_ regulates weight loss and γδ T cell accumulation in adipose tissues of obese mice remains to be further investigated. Bo Hu et al. demonstrated that γδ T cells and adipocyte IL-17RC control fat innervation and thermogenesis 55. Ayano C. *et al* clarified that the important physiological functions for resident γδ T cells in adipose tissue immune homeostasis and body-temperature control 56. These studies indicate that γδ T cells that produce cytokine IL-17 (γδT17) play an important role in regulating infection, inflammation, cancer, and insulin resistance 57-59. We also observed that IL-17A levels in both circulating γδ T cells and adipose tissue residual γδ (Vγ1 and Vγ4) T cells in obese mice were higher than those in normal chow-fed mice, whereas the expression of IL-17A was barely affected by 1α,25(OH)_2_D_3_ treatment. Finally, investigations are needed to fully understand how vitamin 1α,25(OH)_2_D_3_, γδ T cells, obesity, and the inflammation axis affect insulin resistance and T2D.

## Conclusion

In summary, our study revealed that 1α,25(OH)_2_D_3_ directly targeted VDR to promote the expression of FBP1, thereby driving the dephosphorylation of Akt/p38 MAPK and reducing the production of TNF-α and IFN-γ. Administration of 1α,25(OH)_2_D_3_ reduced the expression of inflammatory cytokines of γδ T cells residing in the adipose tissue and ameliorated systemic insulin resistance in obese mice. These findings provide new insights into 1α,25(OH)_2_D_3_ biology and show that 1α,25(OH)_2_D_3_ is a safe compound with promising potential to prevent obesity, inflammation, and insulin resistance.

## Supplementary Material

Supplementary figures and tables.
