# Supplementary material for: 1α,25(OH)2D3 ameliorates insulin resistance by alleviating γδ T cell inflammation via enhancing fructose-1,6-bisphosphatase 1 expression
Source: Theranostics. 2023 Sep 25;13(15):5290–304. doi: 10.7150/thno.84645 (PMC10614678; doi:10.7150/thno.84645)
Supplement: Supplementary file 1 — Supplementary figures and tables. [file thnov13p5290s1.pdf]

Supplementary materials for

1α,25$(OH)_2D_3$ ameliorates insulin resistance by alleviating γδ T cell inflammation via enhancing fructose-1,6-bisphosphatase 1 expression

Table S1. Baseline Characteristics of Patients with T2D

Table S1. Baseline Characteristics of Patients with T2D.

	Group 1 (BMI 18.5-23.9)	Group 2 (BMI 24-27.9)	Group 3 (BMI > 28)
No.	n = 27	n = 25	n = 16
Male: female	15:12	16:9	9:7
Age (years)	55 (42.0-68.0)	66 (45.5-72.5)	64.5 (57.0-70.0)
BMI (kg/m2)	22.1 (21.08-22.48)	25.78 (24.88-27.41)	29.62 (28.94-31.69)
HbA1C (%)	9.6 (6.7-11.6)	7.8 (6.9-9.75)	8.1 (6.3-9.5)
Glucose (mmol/L)	6 (5.0-9.78)	6.54 (5.45-8.365)	7.925 (5.525-10.47)
CR (μmol/L)	90 (76.0-119.0)	110 (86-133.5)	99.5 (84.5-123.8)
ALT (u/L)	13 (9.0-19.0)	16 (10.0-21.0)	20 (13.0-31.75)
AST (u/L)	16 (12.0-20.0)	16 (13.5-19.0)	19 (15.75-23.25)
VA (μmol/L)	1.319 (0.932-1.63)	1.207 (0.7925-1.616)	1.604 (1.141-2.013)
VB1 (nmol/L)	77.75 (57.26-108.8)	83.11 (58.03-106.4)	88.34 (60.72-105.4)
VB9 (nmol/L)	23.29 (20.88-25.92)	22.87 (20.61-24.22)	22.31 (19.75-25.55)
VB12 (pg/mL)	197.3 (194.9-209.0)	197 (194.9-198.5)	196.4 (192.7-259.2)
VC (μmol/L)	70.17 (53.22-90.31)	79.05 (63.17-99.05)	70.92 (59.4-89.81)
VD (nmol/L)	55.42 (24.68-66.66)[a]	44.5 (29.0-53.94)[b]	24.92 (24.06-44.35)
VE (μg/mL)	12.9 (11.09-13.76)	13.26 (12.43-13.9)	13.55 (11.22-14.36)

Data are expressed as medians and interquartile range (Q1-Q3).

[a]Significant difference between Group 1 and Groups 3 ($P < 0.05$).

[b]Significant difference between Group 2 and Groups 3 ($P < 0.05$).

BMI, Body Mass Index; HbA1C, Hemoglobin A1C; CR, creatinine; ALT, alanine aminotransferase; AST, aspartate aminotransferase; VA, vitamin A; VB1, vitamin B1; VB9, vitamin B9; VB12, vitamin B12; VC, vitamin C; VD, 25(OH)D3; VE, vitamin E.

Table S2. Primers used for quantitative RT-PCR

Table S2. Primers used for quantitative RT-PCR; all primers are listed in 5' to 3' sequence.

Primer name	Forward Primer	Reverse Primer
Human PDK3	CGCTCTCCATCAAACAATTCCT	CCACTGAAGGGCGGTTAAGTA
Human DLD	CTCATGGCCTACAGGGACTTT	GCATGTTCCACCAAGTGTTTCAT
Human DLAT	CCGCCGCTATTACAGTCTTCC	CTCTGCAATTAGGTCACCTTCAT
Human IDH3B	GAGCCAAGTCTCAGCGGATT	GGGCATCACAAGCACATCAAA
Human IDH3A	CCCGCGTGGATCTCTAAGG	AATTTCTGGGCCAATACCATCTC
Human ACO2	CCCTACAGCCTACTGGTGACT	TGTACTCGTTGGGCTCAAAGT
Human CS	CGCTACATCATCAATACAGCGG	GCATGGACTTAATGACCACCC
Human MDH2	TCGGCCCAGAACAATGCTAAA	GCGGCTTTGGTCTCGATGT
Human FH	CCTGTGCATCCCAACGATCAT	AATTCCTGCCCAAGAGTAAGTG
Human OGDH	TTGGCTGGAAAACCCCAAAAG	TGTGCTTCTACCAGGGACTGT
Human HIF1A	GTCTGAGGGGACAGGAGGAT	AAAGGCAAGTCCAGAGGTGG
Human C-MYC	GTCAAGAGGCGAACACACAAC	TTGGACGGACAGGATGTATGC
Human GLUT9	CCTCTACGGCTACAACCTGTC	AGAGTGTCTGGGTCTATTGGAC
Human GLUT1	TGGCATCAACGCTGTCTTCT	CTAGCGCGATGGTCATGAGT
Human LDHD	AGGTGCGAACCTCCTGATG	CGGTGCCGAATGGGATGAT
Human LDHB	TGGTATGGCGTGTGCTATCAG	TTGGCGGTCACAGAATAATCTTT
Human FBP1	CGCGCACCTCTATGGCATT	TTCTTCTGACACGAGAACACAC
Human PGM	AGCATTCCGTATTTCCAGCAG	GCCAGTTGGGGTCTCATACAAA
Human PGK1	CATACCTGCTGGCTGGATGG	CCCACAGGACCATTCCACAC
Human LDHA	ATGGCAACTCTAAAGGATCAGC	CCAACCCCAACAACTGTAATCT
Human PKM2	ATGTCGAAGCCCCATAGTGAA	TGGGTGGTGAATCAATGTCCA
Human HK2	GAGCCACCACTCACCCTACT	CCAGGCATTCGGCAATGTG
Human VDR	GTGGACATCGGCATGATGAAG	GGTCGTAGGTCTTATGGTGGG
Human IL-1β	CAGAAGTACCTGAGCTCGCC	AGATTCGTAGCTGGATGCCG
Human IFN-γ	TGAATGTCCAACGCAAAGCA	CTGGGATGCTCTTCGACCTC
Human Granzyme B	CCCTGGGAAAACACTCACACA	GCACAACTCAATGGTACTGTCG
Human TNF-α	CACAGTGAAGTGCTGGCAAC	AGGAAGGCCTAAGGTCCACT
Human IL-17A	AGATTACTACAACCGATCCACCT	GGGGACAGAGTTCATGTGGTA
Human Perforin	GGCTGGACGTGACTCCTAAG	CTGGGTGGAGGCGTTGAAG
Human IL-10	GAGGAAAAAAAATGTTCTTTGGGGA	GGGGCTCCCTGGTTTCTCTTCCTAA

Human IL-15	CATTTTGGGCTGTTTCAGTGC	GCTGTTACTTTGCAACTGGGG
Human IL-2	AACCTCAACTCCTGCCACAA	GCATCCTGGTGAGTTTGGGA
Human IL-23	GAGCAGAGCTGTAATGCTGC	GTGCAGAGCTTCTGTGAAAGC
Human IL-6	TTCGGTCCAGTTGCCTTCTC	TGAGATGCCGTCGAGGATG
Human IL-4	AGCAGTTCCACAGGCACAAG	ACTCTGGTTGGCTTCCTTCAC
Human Fas	GGACCCTCCTACCTCTGGTT	ACCTGGAGGACAGGGCTTAT
Human IL-17F	GCGTTTCCATGTCACGTAACA	CAGCCCAAGTTCCTACACTGG
Human IL-13	CATGGCGCTTTTGTTGACCA	AGCTGTCAGGTTGATGCTCC
Human β-actin	TTCGACAGTCAGCGCATCTTCTT	GCCCAATACGACCAAATCCGTTGA
Mouse FBP1	GCATCGCACAGCTCTATGGT	TGGTTCCGATGGACACAAGG
Mouse GAPDH	AGGTCGGTGTGAACGGATTTG	TGTAGACCATGTAGTTGAGGTCA

Table S3. Primers used for ChIP-PCR

**Table S3. Primers used for ChIP-PCR, all primers are listed in 5' to 3' sequence.**		
**Primer name**	**Forward Primer**	**Reverse Primer**
VDR binding site-1 (FBP1 promoter)	TTCCTGAAAAGTTGCCTGAGGA	TGCCTGGCAAAGAGAGTTGA
VDR binding site-2 (FBP1 promoter)	TCCTAGTAACCTGGAGGGCA	GAGGCATGGTCTACCCTGTG

Table S4. Key Resources Table

**REAGENT**	**SOURCE**	**IDENTIFIER**
**Antibodies**		
Anti-human CD3 (UCHT1)	BD Biosciences	Cat # 561416; RRID: AB_10611584
Anti-human TCR γδ (B1)	BD Biosciences	Cat # 564157; RRID: AB_2738629
Anti-human CD4 (OKT4)	Biolegend	Cat # 317431; RRID: AB_2028492
Anti-human CD8 (HIT8a)	Biolegend	Cat # 300921; RRID: AB_1575076
Anti-human TCR Vδ2 (B6)	Biolegend	Cat # 331410; RRID: AB_1877263
Anti-human TNF-α (Mab11)	Biolegend	Cat # 502909; RRID: AB_315261
Anti-human IFN-γ (4S.B3)	Biolegend	Cat # 502512; RRID: AB_315237
Anti-human/mouse Granzyme B (QA18A28)	Biolegend	Cat # 396409; RRID: AB_2801078
Anti-human Perforin (dG9)	Biolegend	Cat # 308121; RRID: AB_2566203
Anti-human TCR Vδ1 (REAL277)	Miltenyi	Cat # 130-115-979; RRID: AB_2751297
Anti-mouse CD3 (17A2)	Biolegend	Cat # 100220; RRID: AB_1732057
Anti-mouse CD4 (RM4-4)	Biolegend	Cat # 116006; RRID: AB_313691
Anti-mouse CD8 (53-6.7)	Biolegend	Cat # 100732; RRID: AB_893423
Anti-mouse TCR γδ (GL3)	Biolegend	Cat # 118119; RRID: AB_10896753
Anti-mouse TNF-α (MP6-XT22)	Biolegend	Cat # 506304; RRID: AB_315425
Anti-mouse IFN-γ (XMG1.2)	Biolegend	Cat # 505809; RRID: AB_315403
Anti-mouse TCR Vγ1.1/Cr4 (2.11)	Biolegend	Cat # 141109; RRID: AB_2750498
Anti-mouse TCR Vγ2 (UC3-10A6)	Biolegend	Cat # 137707; RRID: AB_10899574
Anti-mouse CD3ε (145-2C11)	Biolegend	Cat # 100325; RRID: AB_893319
Anti-mouse IL-17A (TC11-18H10.1)	Biolegend	Cat # 506941; RRID: AB_2565836
Anti-mouse TCRγ/δ (UC7-13D5)	Biolegend	Cat # 107517; RRID: AB_2813964
Rabbit anti-FBP1 (D2T7F)	Cell Signaling Technology	Cat # 59172S; RRID: AB_2799559
Rabbit anti-VDR (D2K6W)	Cell Signaling Technology	Cat # 12550S; RRID: AB_2637002
Rabbit anti-p-Akt (Ser473)	Cell Signaling Technology	Cat # 4060T; RRID: AB_2315049

Rabbit anti-Akt (C67E7)	Cell Signaling Technology	Cat # 4691; RRID: AB_915783
Rabbit anti-p-p38 MAPK (Thr180/Tyr182)	Cell Signaling Technology	Cat # 4511; RRID: AB_2139682
Rabbit anti-p38 MAPK (D13E1)	Cell Signaling Technology	Cat # 8690T; RRID: AB_10999090
Rabbit anti-p-PDK1 (Ser241)	Cell Signaling Technology	Cat # 3438; RRID: AB_2161134
Rabbit anti-PDK1	Cell Signaling Technology	Cat # 3062; RRID: AB_2236832
Rabbit anti-p-p65 (Ser536)	Cell Signaling Technology	Cat # 3033; RRID: AB_331284
Rabbit anti-p65 (D14E12)	Cell Signaling Technology	Cat # 8242; RRID: AB_10859369
Rabbit anti-p-AMPKα (Thr172)	Cell Signaling Technology	Cat # 2535; RRID: AB_331250
Rabbit anti-AMPKα (D5A2)	Cell Signaling Technology	Cat # 5831T; RRID: AB_10622186
Rabbit anti-p-p70S6K (Thr421/Ser424)	Cell Signaling Technology	Cat # 9204S; RRID: AB_2265913
Rabbit anti-p70S6K (49D7)	Cell Signaling Technology	Cat # 2708; RRID: AB_390722
Mouse anti-β-actin (8H10D10)	Cell Signaling Technology	Cat # 3700; RRID: AB_2242334
Anti-mouse IgG, HRP-linked Antibody	Cell Signaling Technology	Cat # 7076; RRID: AB_330924
Anti-rabbit IgG, HRP-linked Antibody	Cell Signaling Technology	Cat # 7074; RRID: AB_2099233
Normal Rabbit IgG	Cell Signaling Technology	Cat # 2729; RRID: AB_1031062
Goat anti-Mouse IgG, IgM (H+L) Alexa Fluor 488	Thermo Fisher Scientific	Cat # A-10680; RRID: AB_2534062
**Chemicals, peptides, and recombinant proteins**		
Zoledronic acid monohydrate	MedChemExpress	Cat # HY13777A
Calcitriol	MedChemExpress	Cat # HY-10002
D-Glucose	MedChemExpress	Cat # HY-B0389
MB05032	MedChemExpress	Cat # HY-16307
CFSE	MedChemExpress	Cat # HY-D0938
Puromycin dihydrochloride	MedChemExpress	Cat # HY-B1743A
Phorbol 12-myristate 13-acetate	Sigma-Aldrich	Cat # P8139
Ionomycin	Sigma-Aldrich	Cat # I9657
Insulin	Sigma-Aldrich	Cat # I3536
Collagenase Type II	Sigma-Aldrich	Cat # C6885
Triton X−100	Sigma-Aldrich	Cat # V900502
Protease Inhibitor Cocktail	Bimake	Cat # B14001
Phosphatase Inhibitor Cocktail	Bimake	Cat # B15001
1alpha,25-Dihydroxyvitamin D3	Enzo Life Sciences	Cat # BML-DM200-0050
Ficoll-Paque PLUS	GE Healthcare	Cat # 17-1440-03
Fluoroshield mounting medium with DAPI	Abcam	Cat # ab104139
Recombinant human IL-2	Peprotech	Cat # 200-02-100
Adezmapimod (SB203580)	Selleck	Cat # S1076
**Critical commercial assays**		
PrimeScript RT Master Mix	TaKaRa	Cat # RR038B
Lenti-X Concentrator	TaKaRa	Cat # 631231
Lipofectamine 3000 Transfection Reagent	Thermo Fisher Scientific	Cat # L3000015

SimpleChIP® Enzymatic Chromatin IP Kit	Cell Signaling Technology	Cat # 9003S
Glucose (GO) Assay Kit	Sigma-Aldrich	Cat # GAGO20
EasySep™ Human Gamma/Delta T Cell Isolation Kit	STEMCELL Technologies	Cat # 19255
BD PMG Cytofix/Cytoperm Soln Kit	BD Pharmingen	Cat # 554714
Golgi Stop	BD Pharmingen	Cat # 554724
XF Glycolysis Stress Test Kit	Agilent	Cat # 103020-100
XF Cell Mito Stress Test Kit	Agilent	Cat # 103015-100
Human 25-hydroxyvitamin D3 ELISA Kit	MIbio Tech	Cat # ml063235
Mouse 25-hydroxyvitamin D3 ELISA Kit	Jianglaibio Tech	Cat # JL20117
RNAsimple Total RNA kit	Tiangen	Cat # DP419
RIPA Lysis Buffer	Beyotime Biotech	Cat # P0013C
Rodent Diet with 60% kcal% fat	Research Diets	Cat # D12492
2x SYBR Green qPCR Master Mix	Bimake	Cat # B21202
**Deposited data**		
RNA-seq data	This paper	GEO: GSE213910
Listed in Supplementary Table 2 and Table 3	This paper	N/A
**Experimental models: organisms/strains**		
C57BL/6J	Charles River	N/A
**Recombinant DNA**		
lentiCRISPR v2	Addgene	Cat # 52961
lentiCRISPR v2-VDR knock out vector	This paper	NA
psPAX2	Addgene	Cat # 12260
pMD2.G	Addgene	Cat # 12259
**Software and algorithms**		
Flow Jo	Tree Star	Version VX
GraphPad Prism	GraphPad Software	Version 9.0
JASPAR for VDR binding site predication	JASPAR	https://jaspar.genereg.net/
Chord diagram analysis	Bioinformatics	https://www.bioinformatics.com.cn
Heatmap analysis	Heatmap illustrator	http://hemi.biocuckoo.org/faq.php
**Other**		
BD FACSVerse	BD Biosciences	N/A
Blood glucose strips	Johnson	N/A
XF96 Extracellular Flux Analyzer	Agilent	N/A
Bio-Rad ChemiDoc MP Gel imaging system	Bio-Rad	N/A
CFX Connect Real-Time PCR Detection System	Bio-Rad	N/A
Leica TCS SP2 AOBS confocal	Leica Microsystems	N/A
RPMI 1640 Medium	Thermo Fisher Scientific	Cat # 11875093

Supplementary Figures and legends

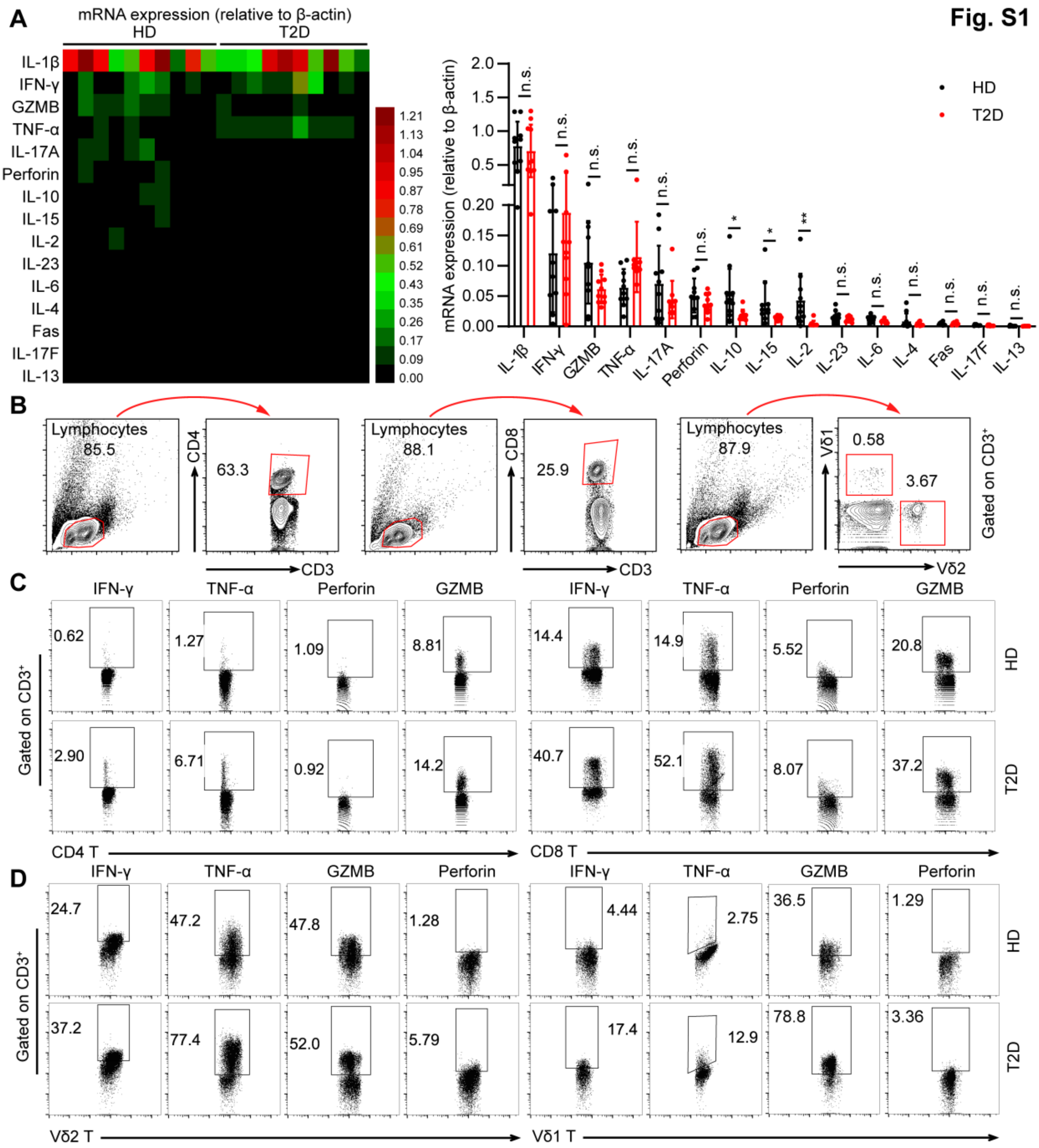

Figure S1. The level of cytokines of T cells in healthy donors and patients with type 2 diabetes. (A) mRNA expression of cytokine genes in circulating γδ T cells was detected by qPCR (HD = 10, T2D = 10). **(B)** Gating strategy of leukocytes. The percentage of $CD4^+$, $CD8^+$, $V\delta2^+$, and $V\delta1^+$ T cells were detected by FACS. **(C** and **D)** IFN-γ, TNF-α, Granzyme B, and Perforin production of T cells (gated on $CD3^+ CD4^+$, $CD3^+ CD8^+$, $CD3^+ V\delta2^+$ and $CD3^+ V\delta1^+$ T cells) from HD and T2D patients after stimulation for 4 h with phorbol 12-myristate 13-acetate (PMA) and ionomycin (Ion). Two-tailed unpaired Student's *t*-test (A); $^*P < 0.05$, $^{**}P < 0.01$, $^{***}P < 0.001$, $^{****}P < 0.0001$. n.s., not significant.

Fig. S2

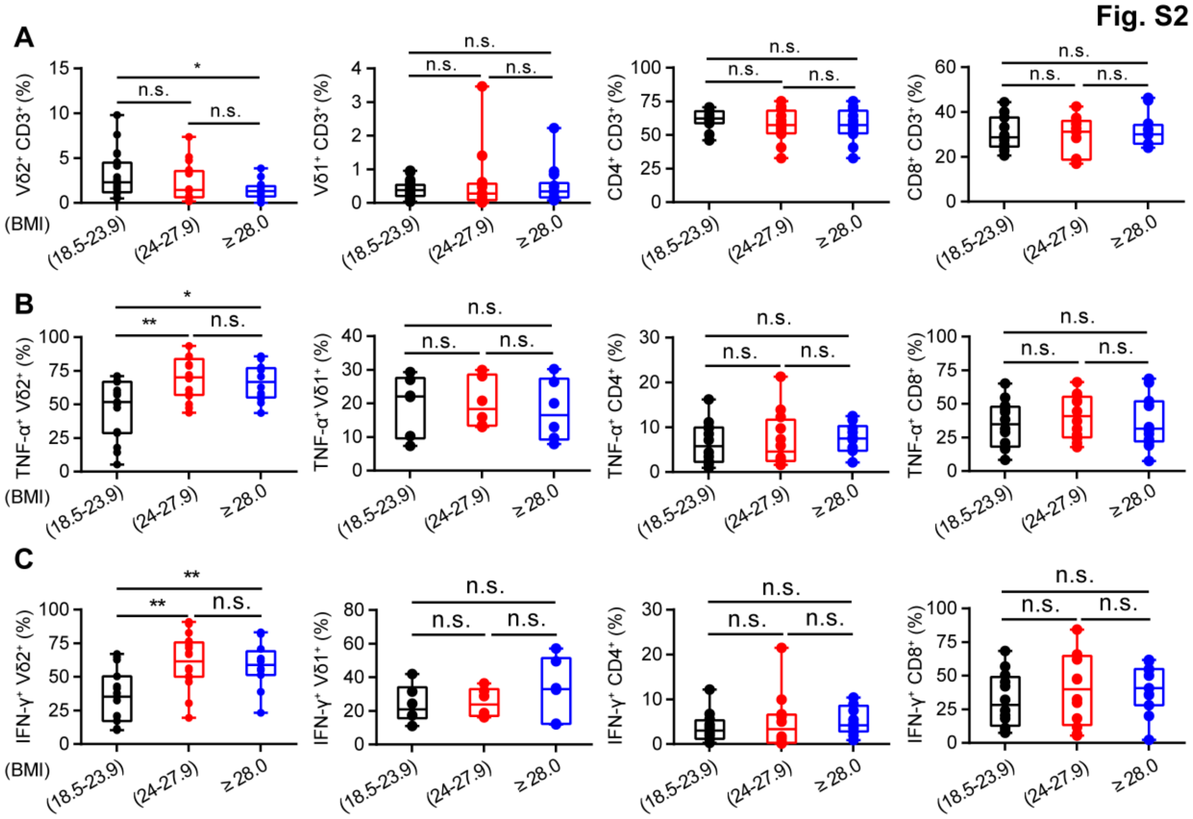
A
B
C
Vδ2⁺ CD3⁺ (%)
Vδ1⁺ CD3⁺ (%)
CD4⁺ CD3⁺ (%)
CD8⁺ CD3⁺ (%)
TNF-α⁺ Vδ2⁺ (%)
TNF-α⁺ Vδ1⁺ (%)
TNF-α⁺ CD4⁺ (%)
TNF-α⁺ CD8⁺ (%)
IFN-γ⁺ Vδ2⁺ (%)
IFN-γ⁺ Vδ1⁺ (%)
IFN-γ⁺ CD4⁺ (%)
IFN-γ⁺ CD8⁺ (%)
(BMI)
(18.5-23.9)
(24-27.9)
≥28.0
n.s.
*
**

Figure S2. Obesity induces cytokine production in circulating Vδ2 T cells from patients with T2D. (A) Box-plot showed the percentage of $V\delta2^+$, $V\delta1^+$, $CD4^+$, and $CD8^+$ T cells of patients with T2D. BMI (kg/m^2) was categorized as healthy weight (18.5-23.9; $V\delta2^+$, n = 20; $V\delta1^+$, n = 20; $CD4^+$, n = 11; $CD8^+$, n = 11), overweight (24-27.9, $V\delta2^+$, n = 17; $V\delta1^+$, n = 17; $CD4^+$, n = 12; $CD8^+$, n = 9), and obesity (≥ 28.0, $V\delta2^+$, n = 14; $V\delta1^+$, n = 14; $CD4^+$, n = 12; $CD8^+$, n = 12). **(B)** TNF-α production of T cells (gated on $CD3^+ CD4^+$, $CD3^+ CD8^+$, $CD3^+ V\delta2^+$ and $CD3^+$ $V\delta1^+$) in patients with T2D. FACS and statistical analysis for the percentage of cytokines. Healthy weight (18.5-23.9; $V\delta2^+$, n = 15; $V\delta1^+$, n = 6; $CD4^+$, n = 12; $CD8^+$, n = 12), overweight (24.0-27.9, $V\delta2^+$, n=16; $V\delta1^+$, n = 6; $CD4^+$, n = 12; $CD8^+$, n = 12), and obesity (≥ 28.0, $V\delta2^+$, n = 12; $V\delta1^+$, n = 6; $CD4^+$, n = 10; $CD8^+$, n = 12). **(C)** IFN-γ production of T cells (gated on $CD3^+$ $CD4^+$, $CD3^+ CD8^+$, $CD3^+ V\delta2^+$ and $CD3^+ V\delta1^+$) in patients with T2D. Healthy weight (18.5-23.9; $V\delta2^+$, n = 15; $V\delta1^+$, n = 6; $CD4^+$, n = 12; $CD8^+$, n = 12), overweight (24.0-27.9, $V\delta2^+$, n = 16; $V\delta1^+$, n = 6; $CD4^+$, n = 12; $CD8^+$, n = 12), and obesity (≥ 28.0, $V\delta2^+$, n = 12; $V\delta1^+$, n = 6; $CD4^+$, n = 12; $CD8^+$, n = 11). One way ANOVA with Tukey's multiple comparisons test (A-C). Data represented mean ± SD. *$P < 0.05$, **$P < 0.01$, ***$P < 0.001$, ****$P < 0.0001$. n.s., not significant.

Fig. S3

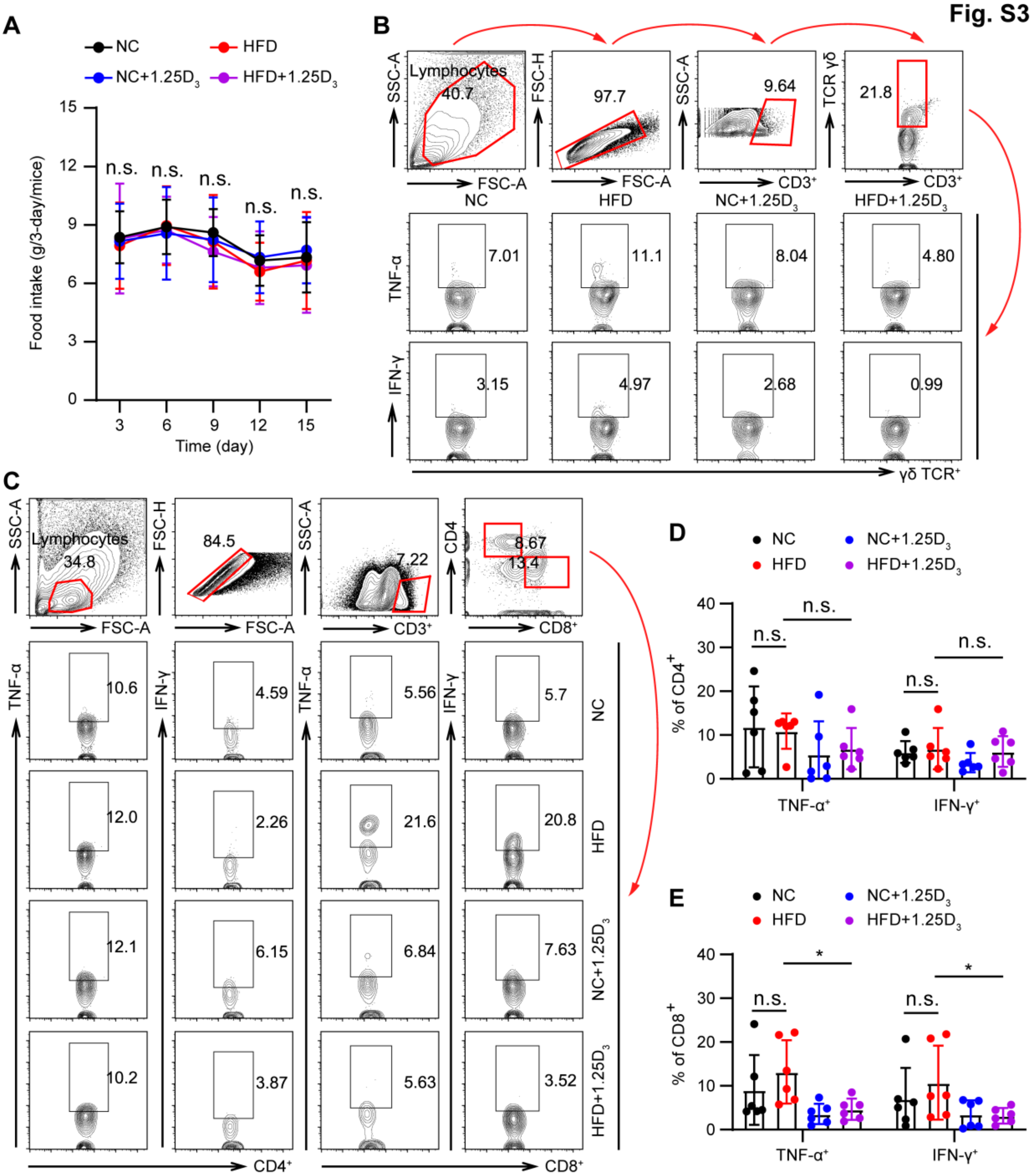

A
NC
HFD
NC+1.25D3
HFD+1.25D3
Food intake (g/3-day/mice)
Time (day)
n.s.
B
Lymphocytes
40.7
97.7
9.64
21.8
SSC-A
FSC-A
FSC-H
CD3+
TCR γδ
NC
HFD
NC+1.25D3
HFD+1.25D3
TNF-α
IFN-γ
7.01
11.1
8.04
4.80
3.15
4.97
2.68
0.99
γδ TCR+
C
Lymphocytes
34.8
84.5
7.22
8.67
13.4
CD4
CD8+
10.6
4.59
5.56
5.7
12.0
2.26
21.6
20.8
12.1
6.15
6.84
7.63
10.2
3.87
5.63
3.52
CD4+
D
% of CD4+
TNF-α+
IFN-γ+
E
% of CD8+
*

Figure S3. Cytokine production of adipose tissue residual T cells reduced after rocaltrol treatment. (A) WT mice were fed with a high fat diet (HFD) or normal chow (ND) for 12 weeks and then treated with rocaltrol (1α,25$(OH)_2D_3$) or PBS for 15 days. Food intake was recorded once every three days (n=3 per group). **(B-E)** Gating strategy of leukocytes. Immune cells in adipose tissue from mice were first gated by FSC-A/SSC-A to exclude debris, followed by gating FSC-A/FSC-H to eliminate non-singlet cells. TCR $\gamma\delta^+$ (B), $CD4^+$ and $CD8^+$ (C) T cells were gated for cytokines (TNF-α and IFN-γ) and statistical analysis (D, E; n = 6 per group). The flow chart represented one example of all samples. One way ANOVA with Tukey's multiple comparisons test (A, D and E). Data represented mean ± SD. *$P < 0.05$, **$P < 0.01$, ***$P < 0.001$, ****$P < 0.0001$. n.s., not significant.

Fig. S4

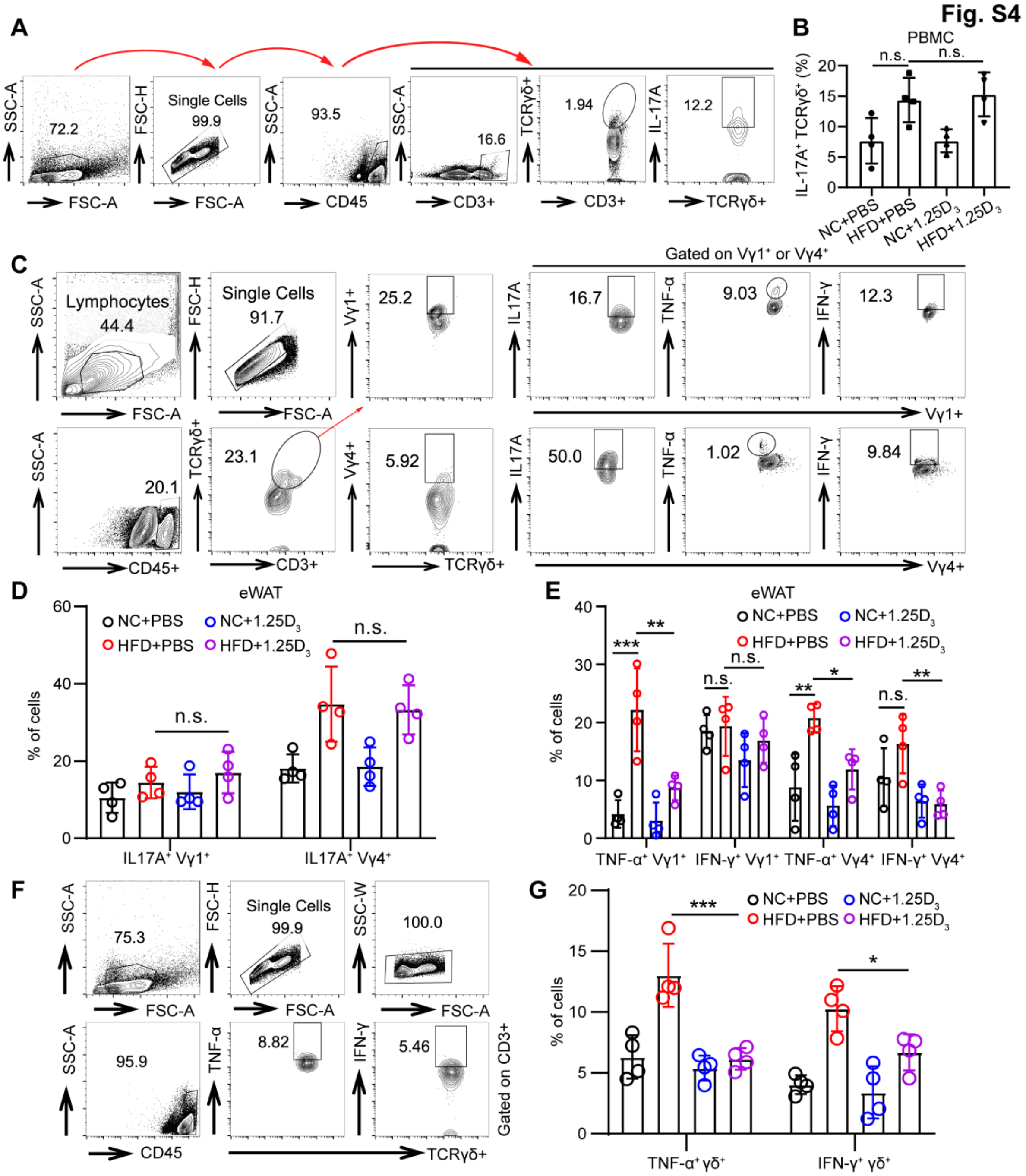

A
SSC-A
FSC-A
72.2
FSC-H
Single Cells
99.9
SSC-A
93.5
CD45
16.6
CD3+
TCRγδ+
1.94
IL-17A
12.2
TCRγδ+
B
PBMC
IL-17A+ TCRγδ+ (%)
n.s.
n.s.
NC+PBS
HFD+PBS
NC+1.25D3
HFD+1.25D3
C
Lymphocytes
44.4
Single Cells
91.7
Vγ1+
25.2
Gated on Vγ1+ or Vγ4+
IL17A
16.7
TNF-α
9.03
IFN-γ
12.3
CD45+
20.1
TCRγδ+
23.1
CD3+
Vγ4+
5.92
50.0
1.02
9.84
D
eWAT
% of cells
NC+PBS
NC+1.25D3
HFD+PBS
HFD+1.25D3
n.s.
IL17A+ Vγ1+
IL17A+ Vγ4+
E
eWAT
TNF-α+ Vγ1+
IFN-γ+ Vγ1+
TNF-α+ Vγ4+
IFN-γ+ Vγ4+
F
75.3
99.9
SSC-W
100.0
95.9
8.82
5.46
Gated on CD3+
G
TNF-α+ γδ+
IFN-γ+ γδ+

Figure S4. The level of cytokine production in adipose tissue residual T cells and peripheral blood mononuclear cell after rocaltrol treatment. (A and **B)** WT mice were fed with a high fat diet (HFD) or normal chow (ND) for 10 weeks and then treated with rocaltrol ($1\alpha,25(OH)_2D_3$) or PBS for 10 days (n = 4 per group). Gating strategy of TCRγδ T cells, immune cells in peripheral blood mononuclear cell from mice were first gated by FSC-A/SSC-A to exclude debris, followed by gating FSC-A/FSC-H to eliminate non-singlet cells (A). Circulating TCR $\gamma\delta^+$ (B) T cells were gated for cytokine (IL-17A$^+$) and statistical analysis (n = 4 per group). **(C-E)** Adipose tissue residual $V\gamma1^+$ and $V\gamma4^+$ T cells were gated for cytokine (IL-17A$^+$, TNF-α^+ and IFN-γ^+) and statistical analysis (n = 4 per group). **(F** and **G)** Circulating TCR $\gamma\delta^+$ T cells were gated for cytokine TNF-α^+ and IFN-γ^+) and statistical analysis (n = 4 per group)**.** The flow chart represented one example of all samples. One way ANOVA with Tukey's multiple comparisons test (B, D, E and G). Data represented mean ± SD. *$P < 0.05$, **$P < 0.01$, ***$P < 0.001$, ****$P < 0.0001$. n.s., not significant.

Fig. S5

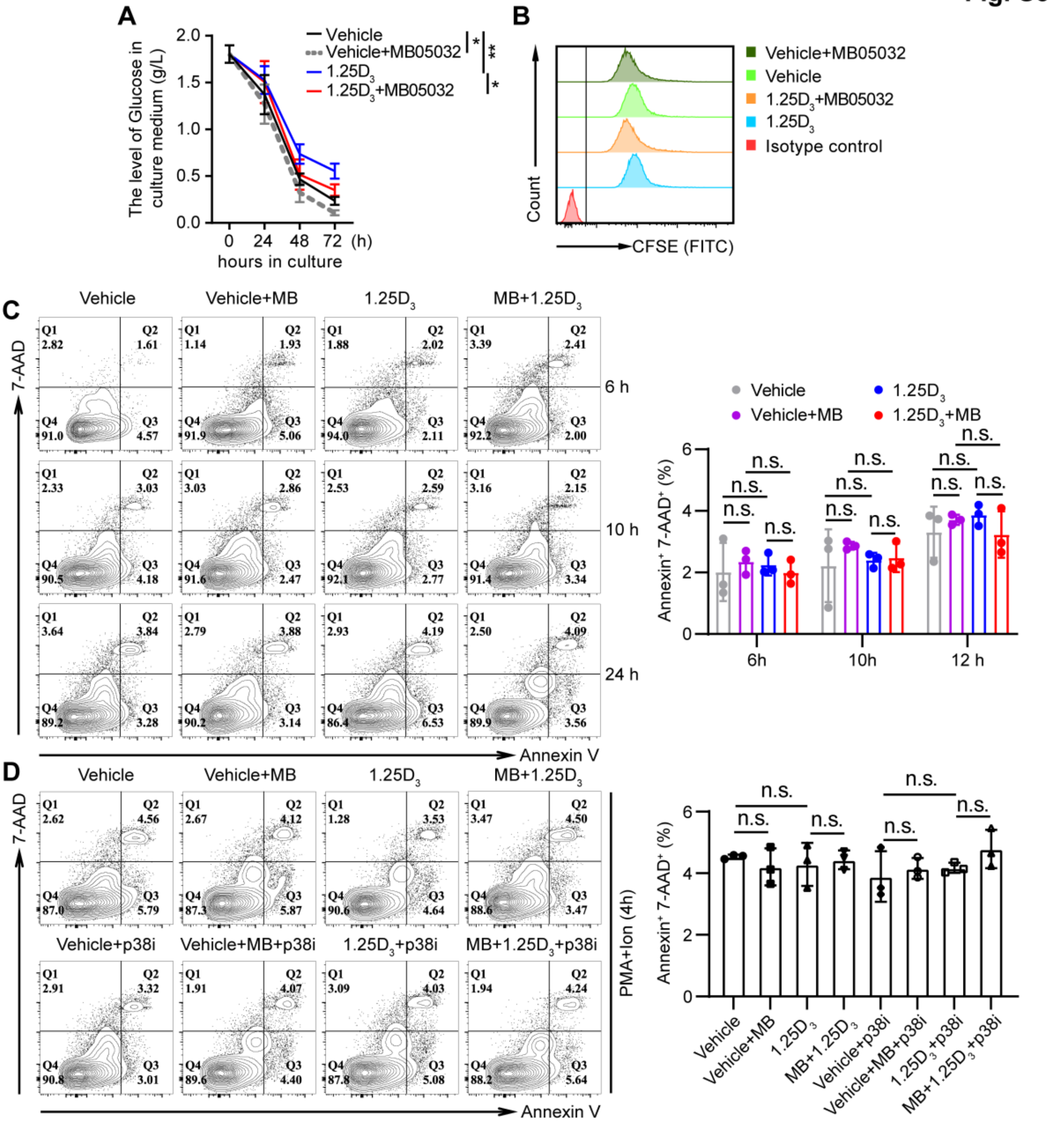

A
The level of Glucose in culture medium (g/L)
hours in culture
Vehicle
Vehicle+MB05032
1.25D3
1.25D3+MB05032
B
Vehicle+MB05032
Vehicle
1.25D3+MB05032
1.25D3
Isotype control
Count
CFSE (FITC)
C
Vehicle
Vehicle+MB
1.25D3
MB+1.25D3
7-AAD
Annexin V
6 h
10 h
24 h
Annexin+ 7-AAD+ (%)
n.s.
D
Vehicle+p38i
Vehicle+MB+p38i
1.25D3+p38i
MB+1.25D3+p38i
PMA+Ion (4h)

Figure S5. 1α,25$(OH)_2D_3$ reduces glucose metabolism in Vδ2 T cells via FBP1. (A) Glucose level in culture medium were analyzed over time for vehicle-, 1α,25$(OH)_2D_3$-, MB05032- or their combination treated groups for 24 h, 48 h, and 72 h (n = 3). **(B)** The proliferation of human Vδ2 T cells was detected at 48 h using CFSE method. **(C)** Vδ2 T cells were incubated with MB05032 (MB), 1α,25$(OH)_2D_3$ (1.25D_3) or vehicle for 6, 10, and 12 hours. **(D)** ZOL expanded Vδ2 T cells were treated with 1α,25$(OH)_2D_3$ or vehicle, followed by restimulation with 1α,25$(OH)_2D_3$, MB05032, or their combination for another 20 h. Then pretreated Vδ2 T cells were stimulated with PMA (50 ng/mL) and Ion (1 μg/mL) with or without for another 4 h. One way ANOVA with Tukey's multiple comparisons test (A, C and D). Data represented mean ± SD. **P* < 0.05, ***P* < 0.01, ****P* < 0.001, *****P* < 0.0001. n.s., not significant.

Fig. S6

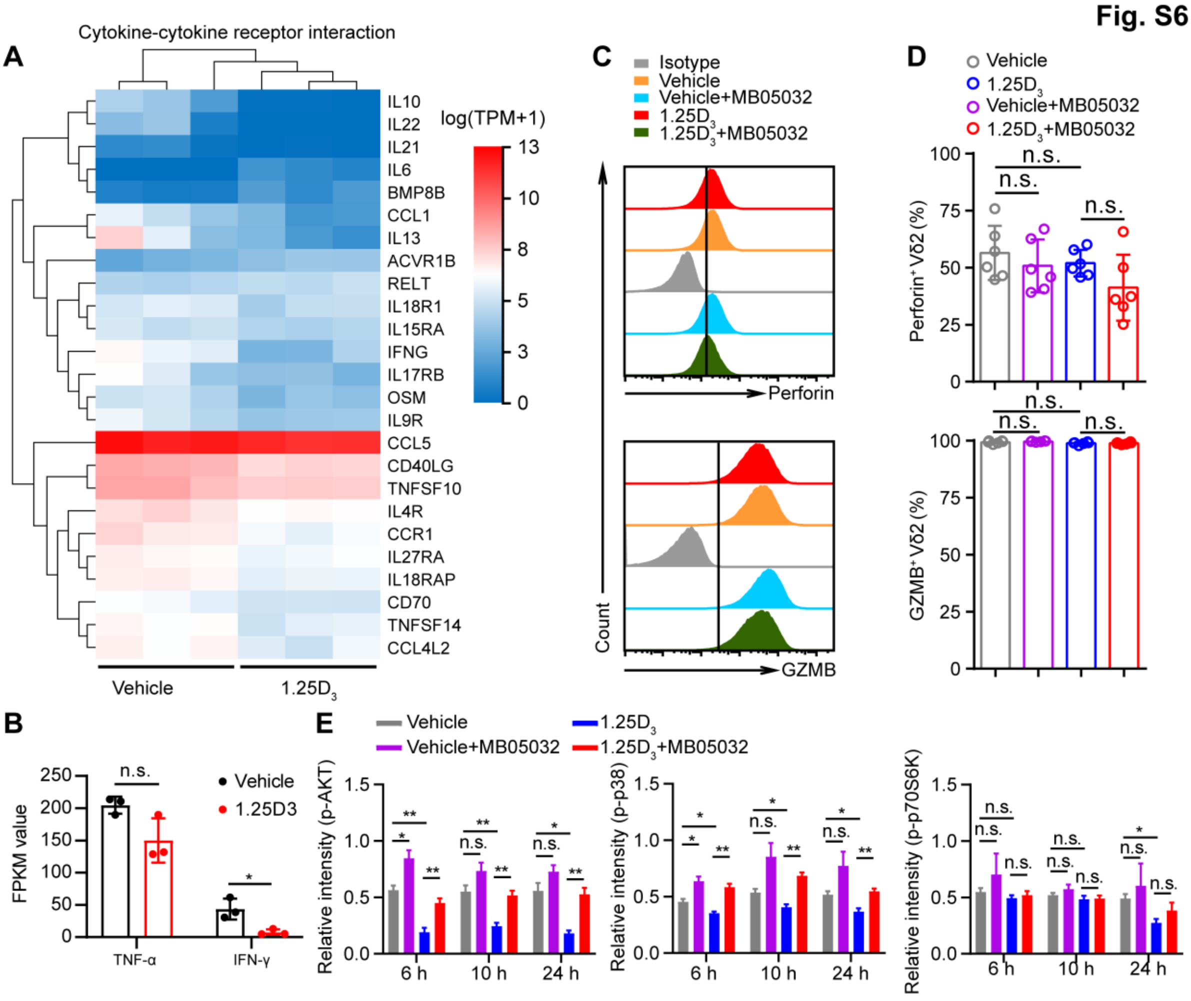
A
Cytokine-cytokine receptor interaction
log(TPM+1)
13
10
8
5
3
0
IL10
IL22
IL21
IL6
BMP8B
CCL1
IL13
ACVR1B
RELT
IL18R1
IL15RA
IFNG
IL17RB
OSM
IL9R
CCL5
CD40LG
TNFSF10
IL4R
CCR1
IL27RA
IL18RAP
CD70
TNFSF14
CCL4L2
Vehicle
1.25D3
B
FPKM value
n.s.
*
Vehicle
1.25D3
TNF-α
IFN-γ
C
Isotype
Vehicle
Vehicle+MB05032
1.25D3
1.25D3+MB05032
Count
Perforin
GZMB
D
Vehicle
1.25D3
Vehicle+MB05032
1.25D3+MB05032
Perforin+ Vδ2 (%)
GZMB+ Vδ2 (%)
n.s.
E
Vehicle
Vehicle+MB05032
1.25D3
1.25D3+MB05032
Relative intensity (p-AKT)
Relative intensity (p-p38)
Relative intensity (p-p70S6K)
6 h
10 h
24 h

Figure S6. FBP1 expression mediated by 1α,25(OH)$_2$D$_3$ do not affect production of perforin and granzyme B of Vδ2 T cells. (A and B) FPKM value of TNF-α and IFN-γ gene (A). Differentially expressed genes from cytokine-cytokine receptor interaction KEGG pathway were presented by heatmap (B) (n = 3). **(C and D)** Vδ2 T cells were treated with 1α,25(OH)$_2$D$_3$ or vehicle, followed by restimulation with 1α,25(OH)$_2$D$_3$, MB05032 or their combination for another 20 h. Then pretreated Vδ2 T cells were stimulated with PMA (50 ng/mL) and Ion (1 μg/mL) for 4 h. FACS and statistical analysis for the percentage of Perforin and Granzyme B were shown (n = 6 per group). (E) Western blot analysis the relative expression of p-Akt, p-p38 and p-p70S6K (n = 3). Two-tailed unpaired Student's *t*-tests (B). Two-tailed unpaired Student's *t*-test (B); One way ANOVA with Tukey's multiple comparisons test (D and E). Data represented mean ± SD. $*P < 0.05$, $**P < 0.01$, $***P < 0.001$, $****P < 0.0001$. n.s., not significant.
